# ARTEMIS: a method for topology-independent superposition of RNA 3D structures and structure-based sequence alignment

**DOI:** 10.1093/nar/gkae758

**Published:** 2024-09-11

**Authors:** Davyd R Bohdan, Janusz M Bujnicki, Eugene F Baulin

**Affiliations:** International Institute of Molecular and Cell Biology in Warsaw, Warsaw, Poland; International Institute of Molecular and Cell Biology in Warsaw, Warsaw, Poland; International Institute of Molecular and Cell Biology in Warsaw, Warsaw, Poland

## Abstract

Non-coding RNAs play a major role in diverse processes in living cells with their sequence and spatial structure serving as the principal determinants of their function. Superposition of RNA 3D structures is the most accurate method for comparative analysis of RNA molecules and for inferring structure-based sequence alignments. Topology-independent superposition is particularly relevant, as evidenced by structurally similar RNAs with sequence permutations such as tRNA and Y RNA. To date, state-of-the-art methods for RNA 3D structure superposition rely on intricate heuristics, and the potential for topology-independent superposition has not been exhausted. Recently, we introduced the ARTEM method for unrestrained pairwise superposition of RNA 3D modules and now we developed it further to solve the global RNA 3D structure alignment problem. Our new tool ARTEMIS significantly outperforms state-of-the-art tools in both sequentially-ordered and topology-independent RNA 3D structure superposition. Using ARTEMIS we discovered a helical packing motif to be preserved within different backbone topology contexts across various non-coding RNAs, including multiple ribozymes and riboswitches. We anticipate that ARTEMIS will be essential for elucidating the landscape of RNA 3D folds and motifs featuring sequence permutations that thus far remained unexplored due to limitations in previous computational approaches.

## Graphical Abstract

**Graphical Abstract:**
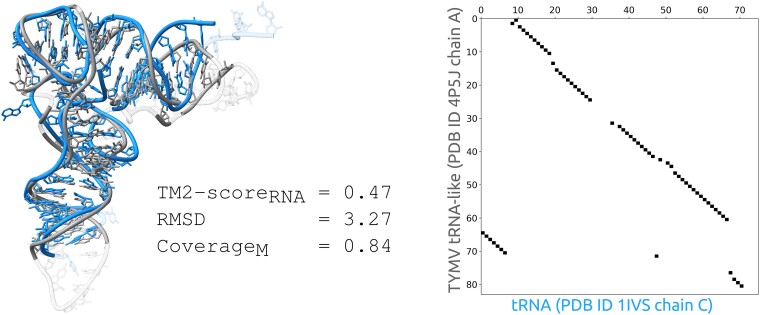

## Introduction

Non-coding RNAs play a major role in diverse processes in living cells with their sequence and spatial structure serving as the principal determinants of their function (1). The rigid-body superposition of RNA 3D structures stands as a fundamental method for the comparative analysis of RNA molecules (2). By superimposing structures of homologous RNAs, it becomes possible to discern conserved and variable segments (3), which is commonly used in template-based RNA structure modeling (4). The 3D superposition of distinct structures of RNA molecules proves beneficial in studies of functionally-related dynamics, such as those between different stages of self-splicing of introns (5) or between apo- and holo- states of riboswitches (6). Furthermore, RNA 3D structure superposition is invaluable for inferring correct alignments (7), given that RNA exhibits significantly less conservation at the sequence level compared to its structural conservation (8). Although flexible superposition is superior for deriving alignments (9), it poses a more challenging task; hence, rigid-body 3D structure alignment remains the gold standard approach (2).

Besides comparisons centered on sequentially-ordered RNA molecules, comparisons have to take into account molecules that are related but do not keep the same structure topology and exhibit sequence permutations: several viral and bacterial RNAs have been identified to mimic tRNA structures, exhibiting both circularly and non-circularly permuted sequence matchings (10,11). Circular permutations have been observed in various natural non-coding RNAs (12,13). Recently, a natural non-circularly permuted variant of a non-coding RNA, specifically the hammerhead ribozyme, was reported (14). Moreover, topology-independent superposition proves valuable for analyzing G-quadruplexes, which adopt diverse backbone topologies in both RNA and DNA (15,16), given the rapidly increasing number of discoveries revealing their relevance in human diseases and therapeutics (17,18).

While several methods, such as Rclick (7) and a more recent development RNAhugs (19), demonstrate capability in topology-independent superposition, comprehensive benchmarking of their performance in this regard has not been undertaken, to the best of our knowledge. Moreover, US-align, standing as the state-of-the-art tool for sequentially-ordered superposition, also exhibits proficiency in topology-independent superposition, although this aspect was not addressed in the original paper (2). The greatest challenge of the RNA 3D structure alignment problem, both sequentially-ordered and topology-independent, is the exponential time complexity of the computational problem (2). Currently, this is being handled either by employing fast but simple heuristics (2) or by relying on intricate but slow ones (19). Furthermore, the problem of detecting backbone-permuted RNA 3D structure similarities has not been previously explored.

Recently, we developed the ARTEM method for unrestrained pairwise superposition of arbitrary RNA 3D modules (20). In this work, we present ARTEMIS (using ARTEM to Infer structure-based Sequence alignments), an application of ARTEM methodology to address the global RNA 3D structure alignment problem. The heuristic behind ARTEMIS operates in polynomial time and ensures the optimal solution, provided it includes at least one residue-residue match with a near-zero RMSD, a condition commonly met in RNA structures due to their characteristic recurrent interactions (20). ARTEMIS significantly outperforms state-of-the-art tools in both sequentially-ordered and topology-independent RNA 3D structure superposition. Leveraging ARTEMIS, we discovered a helical packing motif to be preserved in different backbone topology contexts in diverse non-coding RNAs, including multiple ribozymes and riboswitches.

## Materials and methods

### RNA 3D structure alignment problem

We define the computational problem of pairwise RNA 3D structure alignment as follows. Let *X* and *Y* denote two RNA structures of *N* and *M* residues, respectively. Without loss of generality, we assume *N* ≥ *M* and consider *X* as the static reference structure, with *Y* serving as the query structure undergoing superposition on *X*. An alignment (or superposition) between *X* and *Y* is then defined as a subset of residue pairs (*X*_i_, *Y*_j_), with the condition that each residue can only have one match at most. If an alignment includes at least two pairs of residues (*X*_i_, *Y*_j_) and (*X*_k_, *Y*_l_) such that *i < k* and *j > l*, it is termed a *topology-independent alignment*. In other words, it represents a superposition where residue-residue matchings cannot be sorted monotonically for both sequences simultaneously. Conversely, if such a condition is not met, the superposition denotes *a sequentially-ordered alignment*, or simply a sequence alignment in the traditional sense.

Among all possible alignments, our focus lies on identifying the alignment that captures the most structurally similar subset of residues between *X* and *Y* while achieving the highest possible *coverage*, defined as the ratio of aligned residues to sequence length. In this context, structural similarity refers to the resemblance between rigid coordinate models, commonly assessed through the *root mean square deviation* (*RMSD*) (21). TM-Score metric (22) for proteins and its variant TM-Score_RNA_ (23) for RNAs enable the consideration of both coverage and structural similarity.

TM-Score_RNA_ positively depends on the L_ali_ length of the alignment (the number of matched residue pairs), inversely depends on the *d_i_* distances between the *C3′* atoms of the matched residues, and is normalized by the length *L* of one of the chains, ensuring that the score falls within the range of zero to one (2). For each superposition, two TM-Score_RNA_ values can be computed: TM1-Score_RNA_, normalized by the longer chain length N, and TM2-Score_RNA_, normalized by the shorter chain length M. Likewise, two coverage values are provided for each superposition: L_ali_ / N and L_ali_ / M.

We define the pairwise RNA 3D structure alignment problem as the task of identifying the optimal alignment, whether sequentially-ordered or with potential sequence permutations (topology-independent), which maximizes the sum of (*TM1-Score_RNA_* + *TM2-Score_RNA_*) values.

Drawing from the well-established field of protein bioinformatics, where a 3D fold is defined as the spatial arrangement of all secondary structure elements without considering the sequential order or the connecting loops, in this work we employ a similar conceptual framework for RNA molecules. Here, the RNA 3D fold emphasizes the spatial arrangement of sequence segments in RNA, involved in base-paired structured motifs, primarily helices, while disregarding the order in which these paired sequence segments appear or the nature of the intervening sequences that link them. A common 3D fold implies geometric similarity of RNA structures, without implying the same topology of the chain. Molecules exhibiting the same 3D fold may be related evolutionarily (homologs) or may arise through convergent evolution (analogs).

### ARTEMIS algorithm

To identify the optimal alignment, we developed the ARTEMIS algorithm, which operates under the assumption that the ideal superposition involves at least one pair of matched residues exhibiting a near-zero RMSD.

ARTEMIS superimposes the query structure *Y* on the reference *X* across all possible residue pairs between the structures (Supplementary Figure S1, Supplementary Figure S2, lines 20–32). Each superposition is computed using the Kabsch algorithm (21), employing a 3-atom representation of the residues. This representation comprises three pseudo atoms corresponding to the position of the phosphate group (center of mass of OP1 and O5′ atoms), the ribose (center of mass of *C2′* and *C4′* atoms), and the base (glycosidic base atom). These pseudo atoms were selected among alternatives during preliminary experiments on 500 RNA structure pairs randomly selected among structures of length under 300 nts of the benchmark dataset described below (Supplementary Table S1). For each superposition, ARTEMIS identifies the *hit*, which represents the set of pairs of mutually closest residues between the structures, with the distance between their *C3′* atoms falling below the *MATCHRANGE1* threshold of *3.5 Å* (Supplementary Figure S2, lines 26–29).

For the *TOPLARGEST = M* largest hits, ARTEMIS constructs extended matchings (Supplementary Figure S2, lines 33–53). For each hit, the query structure *Y* undergoes re-superimposition on *X* using the 3-atom representations of the hit's matched residues. Subsequently, the adjusted superposition is utilized to identify an extended set of mutually closest residues between *X* and *Y*, employing the *MATCHRANGE2* threshold of *8 Å* (Supplementary Figure S2, lines 40–43). The resultant matching serves as a candidate topology-independent alignment.

**Figure 1.**
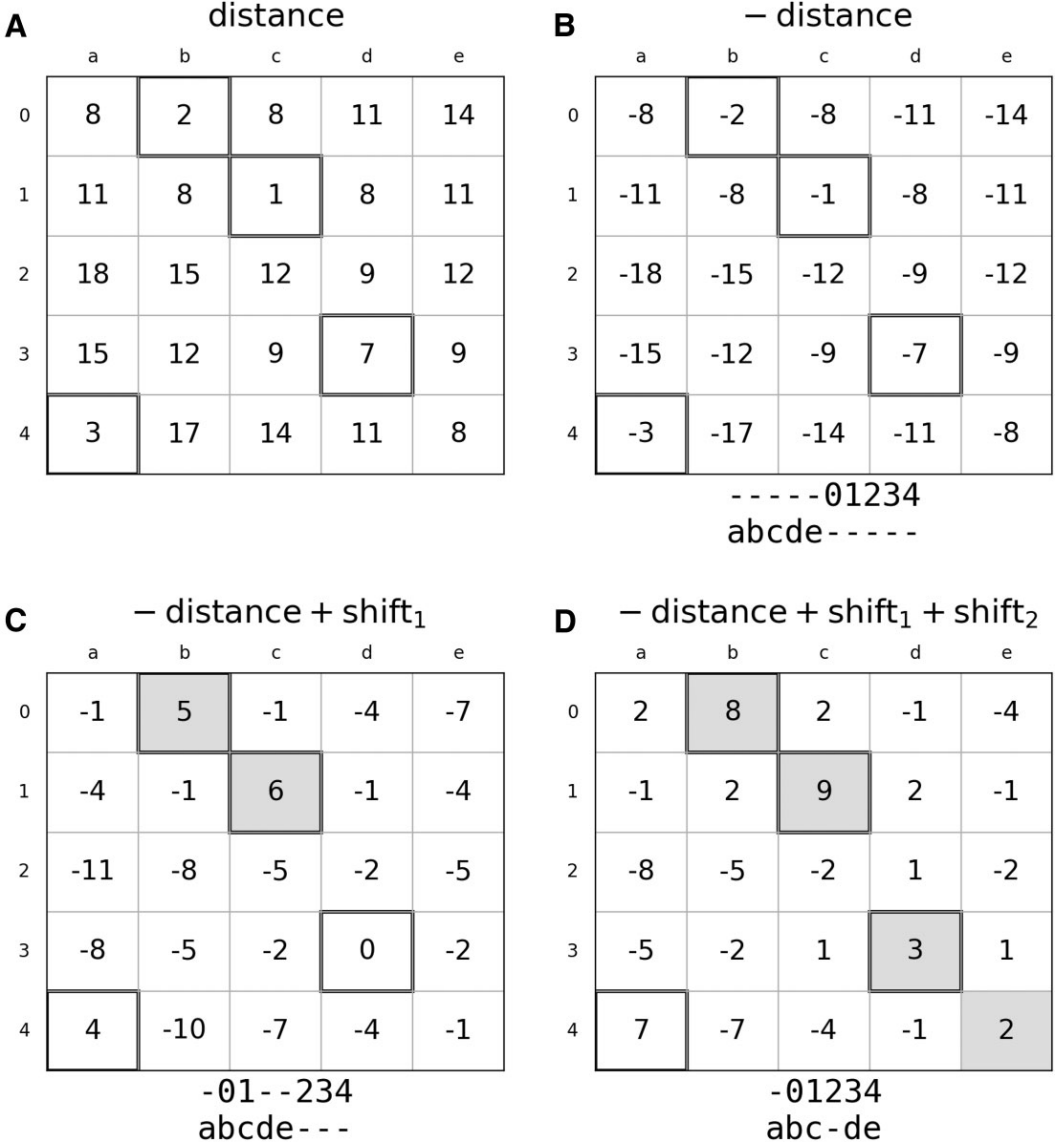
A demonstration example of the scoring matrix preparation procedure used in ARTEMIS for constructing sequentially-ordered alignments. (**A**) A matrix of C3′-C3′ distances. (**B**) The matrix of negative distances. (**C**) The matrix after adding the SHIFT1 term. (**D**) The matrix after adding the SHIFT2 term. Solid-framed cells denote the initial topology-independent hit, while gray-filled cells indicate sequentially-ordered alignments, visually represented as gapped lines below each matrix.

The same adjusted superposition is subsequently utilized to determine the sequentially-ordered alignment (Supplementary Figure S2, lines 45–49). For this purpose, a scoring matrix for the Needleman-Wunsch global sequence alignment algorithm (24) is prepared using the matrix of pairwise C3′-C3′ distances (Figure 1A). This matrix is constructed by negating the distance values (Figure 1B) and then adding the largest distance between the matched residues, denoted as *SHIFT1*, to all values in the negative matrix (Figure 1C), along with an additional term *SHIFT2* = 3 Å (Figure 1D). Subsequently, the Needleman-Wunsch algorithm derives the optimal sequence alignment to maximize the score. A higher *SHIFT2* parameter results in increased coverage, while a *SHIFT2* value of 0 Å ensures that the sequentially-ordered alignment remains a subset of the corresponding topology-independent alignment.

ARTEMIS ultimately provides the optimal sequentially-ordered alignment alongside the optimal topology-independent alignment that encompasses sequence permutations (Supplementary Figure S2, lines 54–55). It's known (2) that, for a given alignment, the minimum-RMSD superposition obtained through the Kabsch algorithm does not typically maximize the TM-Score_RNA_ value. Consequently, for each candidate alignment, ARTEMIS iteratively splits L_ali_ matched residue pairs into fragments of lengths L_ali_ / 2, L_ali_ / 4, …, 3. It proceeds to superimpose each fragment utilizing the Kabsch algorithm, selecting residue pairs whose C3′-C3′ distance value falls below d_0_(M). Subsequently, the Kabsch superposition of all matched residue pairs is executed based on the selected pairs, and the value of *(TM1-Score_RNA_ + TM2-Score_RNA_)* is calculated. The *TM1-Score_RNA_* and *TM2-Score_RNA_* yielding the maximum sum are then assigned to the candidate alignment.

Similar to ARTEM (20), the theoretical time complexity of ARTEMIS is O(N^2^ * M^2^), given that ARTEMIS computes N*M pairwise distances to determine the set of mutually closest residues for each of the N*M single-residue seed superpositions (Supplementary Figure S2, lines 20–30).

### ARTEMIS algorithm acceleration

To expedite ARTEMIS when the length *M* of the smaller structure *Y* exceeds 500 nucleotides, a faster heuristic is employed. Initially, we constrain the total number of single-residue seed superpositions. Instead of considering all N*M possible pairs, ARTEMIS traverses only each *STEP = 1 + M // STEPDIV* residue of *X*, with *STEPDIV = 100* by default. Additionally, we adjust the *TOPLARGEST* value to twice the number of machine CPUs utilized by the ARTEMIS program. Furthermore, we elevate the *SHIFT2* value to *20 Å*. This adjustment does not impact speed but enhances alignment coverage by better capturing related but flexible regions between two long chains within the Needleman-Wunsch alignment. These modifications enable ARTEMIS to process large RNA chains efficiently while maintaining alignment quality.

### ARTEMIS tool implementation

ARTEMIS was implemented in Python3 as a command line interface (CLI) application and is available at https://github.com/david-bogdan-r/ARTEMIS. It requires two DNA- or RNA-containing files in PDB or mmCIF format as input, along with optional arguments, including *MATCHRANGE, TOPLARGEST, SHIFT2*, and *STEPDIV* as described above. By default, ARTEMIS considers all nucleic acid residues from the first models of the input files, unless the user specifies the residues of interest explicitly. The standard output of ARTEMIS comprises information on the input structures, pairwise sequence alignment, and essential alignment quality metrics such as TM-Score_RNA_, RMSD, and alignment length L_ali_. Details of topology-independent alignment are included in the output either upon user request or if its TM2-Score_RNA_ surpasses that of the sequentially-ordered alignment by at least 10%. Optionally, users can save the coordinates of the superimposed query structure along with a list of matched residues and the distances between them for either alignment. Non-default modes of operation include a superposition-only (*-superonly*) mode and an additional-hit (*-addhits*) mode. In the *-superonly* mode, ARTEMIS assumes a trivial alignment between the input structures of equal lengths, which can be used to assess conformational changes between different models of a given RNA. In the *-addhits* mode, ARTEMIS outputs alternative sub-optimal matches above a given threshold, which can be used to find multiple instances of the smaller input structure within the larger one, e.g. to superimpose a tRNA with A- P- and E-state tRNAs of a ribosomal complex in a single run. ARTEMIS is designed as a parallelized program and utilizes all available processors on the machine by default.

ARTEMIS is available to be adapted as a Python3 module, facilitating its integration into environments such as Jupyter Notebooks (https://github.com/david-bogdan-r/ARTEMIS/blob/main/demo.ipynb). Independent implementations of the RNA structure class and the primary ARTEMIS procedure class enable users to bypass unnecessary preliminary procedures, particularly when aligning multiple query structures to a single reference.

### Benchmarking

The performance evaluation of ARTEMIS was based on a dataset comprising 637 RNA chains (2), previously employed for benchmarking US-align (2), RMalign (25), STAR3D (26), ARTS (27) and Rclick (7). All program runs were conducted on an AMD Ryzen 9 5950X machine with 32 CPU cores and 128Gb RAM. We used the default ARTEMIS setting for all benchmarks, which automatically turns on the accelerated mode if the smaller input structure exceeds 500 nucleotides. To compare sequentially-ordered alignments, we conducted superpositions by ARTEMIS (version 1.5), US-align (version 20220227), and Rclick (tar.gz archive dated 22 November 2012) and utilized the alignment quality metrics values of the other tools from the prior benchmark (2). The results of US-align and Rclick obtained by us and those from the previous benchmark were verified to be identical (Supplementary Figure S3). Assessment of sequentially-ordered superposition quality was performed on 168916 structure pairs, as for the remaining 33 650 pairs, at least one of the tools failed to provide a superposition (2).

For topology-independent alignment comparisons, we utilized standalone versions of US-align, Rclick, and RNAhugs (Supplementary Table S2). For RNAhugs, we considered separately the geometric (GEOS) and genetic (GENS) modes. Both GEOS and GENS inherently perform topology-independent superposition by default. Due to RNAhugs's potentially lengthy runtime, which can be tens of seconds even for a superposition of two RNAs under 100 residues, we excluded it from the primary benchmark. As Rclick and RNAhugs do not provide *TM-Score_RNA_* values, we directed their superpositions to US-align to calculate the values without re-superimposing the structures (Supplementary Table S2). Subsequently, we considered RMSD, L_ali_, and TM-Score_RNA_ values as reported in US-align outputs. Evaluation of topology-independent superposition quality was conducted on all 202566 structure pairs.

For each benchmark, we also included the ‘best competitor’ column, determined by selecting, for each structure pair, the alignment with the highest combined (*TM1-Score*_RNA_*+ TM2-Score*_RNA_) value among all tools except ARTEMIS. To evaluate the statistical significance of performance differences among the tools, we employed the paired Student's *t*-test (https://docs.scipy.org/doc/scipy/reference/generated/scipy.stats.ttest_rel.html) and Wilcoxon signed-rank test (https://docs.scipy.org/doc/scipy/reference/generated/scipy.stats.wilcoxon.html), implemented in the SciPy Python library.

To identify similar folds exhibiting sequence permutations, we compared sequentially-ordered and topology-independent superpositions generated by ARTEMIS. We defined a pair of structures to exhibit a backbone-permuted similarity if the topology-independent TM1-Score_RNA_ value was higher than 0.45 and exceeded the sequentially-ordered TM1-Score_RNA_ value by at least 0.1.

To search for minor-groove/minor-groove helical packing motif instances we run ARTEMIS in the additional-hit mode reporting all sub-optimal matches with TM2-Score_RNA_ > 0.3 (Supplementary Table S2).

## Results

### Benchmarking ARTEMIS performance

**Figure 2.**
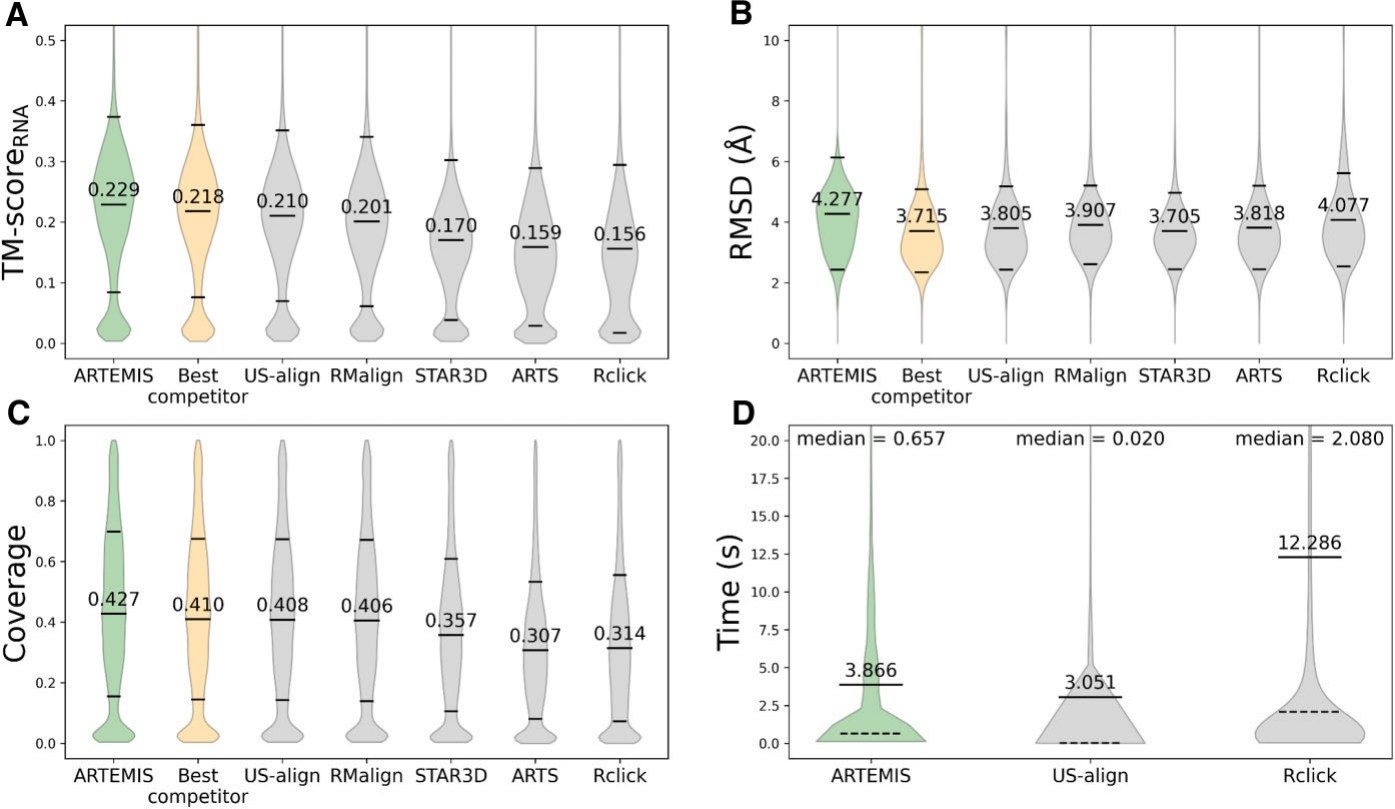
Sequentially-ordered alignment benchmark results. Performance metrics include (**A**) TM-Score_RNA_, (TM1-Score_RNA_ and TM2-Score_RNA_ values together) (**B**) RMSD, (**C**) coverage (coverage1 and coverage2 values together), and (**D**) execution time. ARTEMIS demonstrates superior performance in terms of TM-Score_RNA_, while reporting slightly higher RMSD values, attributed to higher coverage values. Mean ± standard deviation values are specified with dashes, and execution time median values are shown with dashed lines and specified above the violins. Time measurements were conducted for ARTEMIS, US-align and Rclick exclusively, as the results of other tools were directly obtained from the previous benchmark (2).

**Figure 3.**
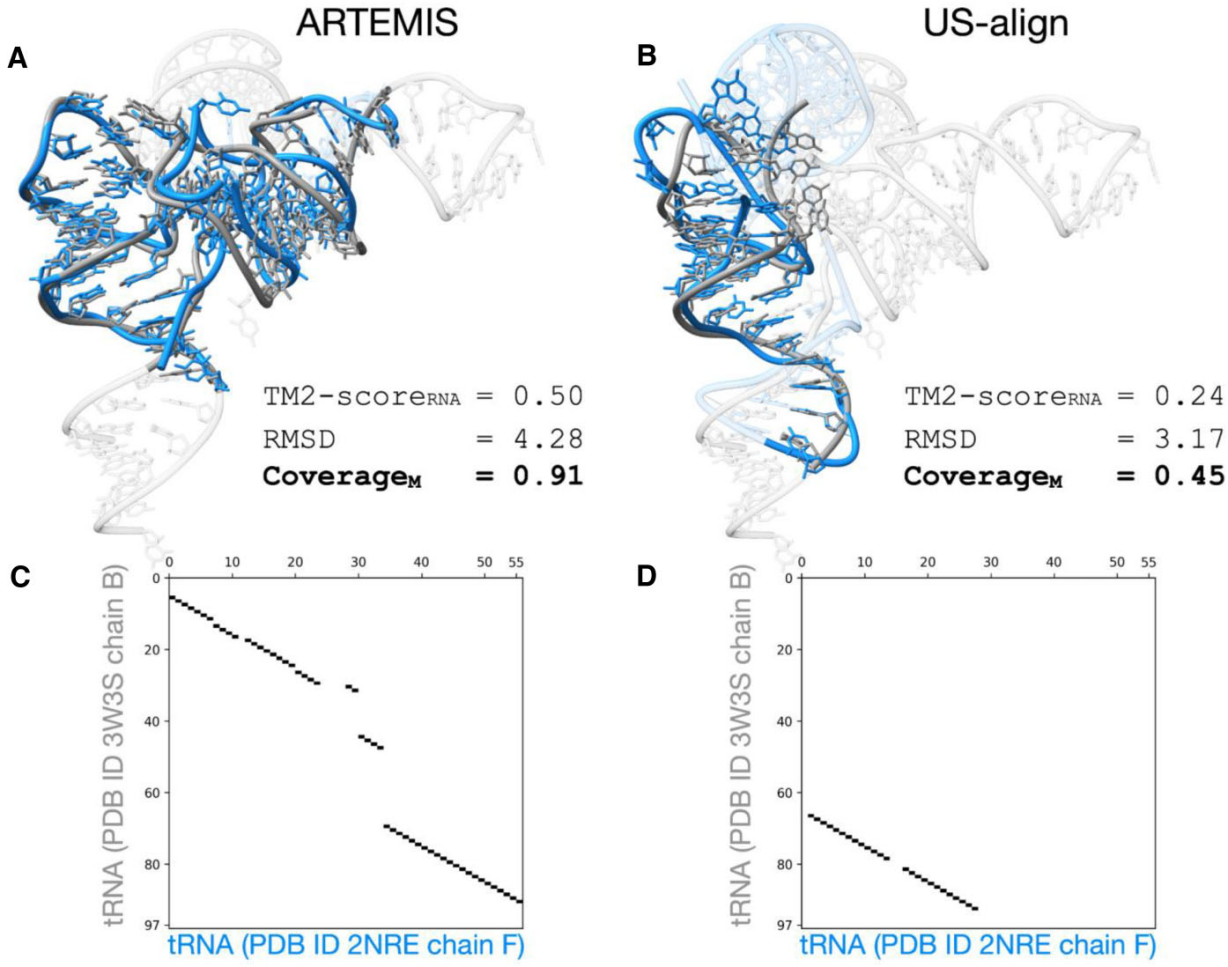
A demonstration example showcasing ARTEMIS' superior performance (**A**, **C**) compared to US-align (**B, D**). Panels C and D depict alignment plots corresponding to the structure superpositions shown in panels A and B, respectively.

**Figure 4.**
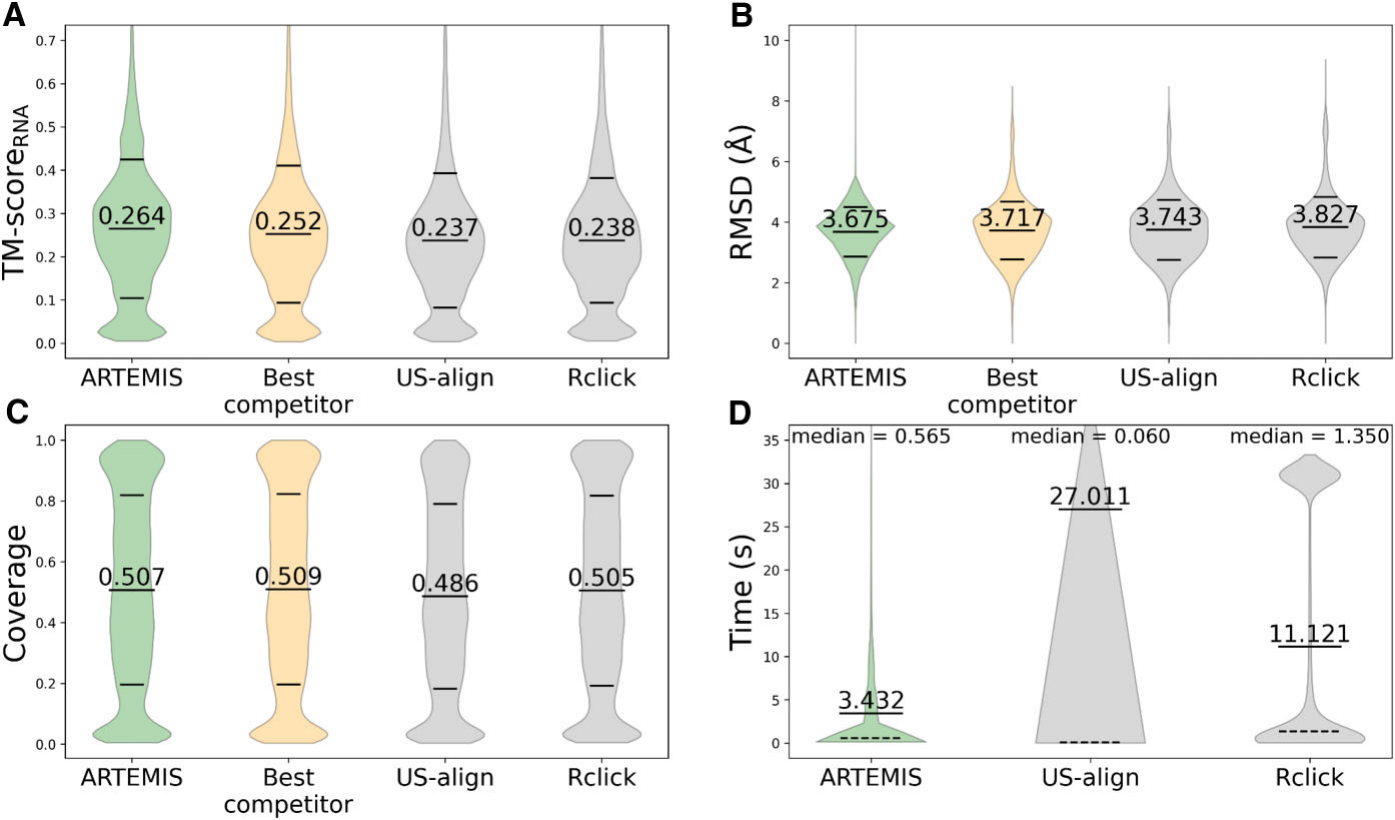
Topology-independent alignment benchmark results. Performance metrics include (**A**) TM-Score_RNA_ (TM1-Score_RNA_ and TM2-Score_RNA_ values together), (**B**) RMSD, (**C**) coverage (coverage1 and coverage2 values together), and (**D**) execution time. ARTEMIS demonstrates superior performance compared to existing tools in terms of TM-Score_RNA_, with lower RMSD values and higher coverage values. Mean ± standard deviation values are specified with dashes, and execution time median values are shown with dashed lines and specified above the violins.

We compared ARTEMIS with state-of-the-art tools in their performance on the curated dataset of 637 RNA chains previously utilized for benchmarking US-align. ARTEMIS significantly outperformed the other tools in terms of TM-*Score_RNA_* values, both in constructing sequentially-ordered alignments (Figures 2, 3, Supplementary Table S3) and topology-independent alignments (Figure 4, Supplementary Table S4).

Compared to US-align, thus far the gold standard, ARTEMIS constructed sequentially-ordered alignments with superior TM-Score*_RNA_* values (mean 0.229 versus mean 0.21, Figure 2A) and reported slightly higher RMSD values (mean 4.277 Å vs. mean 3.805 Å, Figure 2B) attributable to higher coverage values (mean 0.427 versus mean 0.408, Figure 2C). Although US-align operates faster than ARTEMIS in the sequentially-ordered alignment mode (mean 3.051 s versus mean 3.866 s, Figure 2D), ARTEMIS still required under four seconds on average, with the maximum time for a superposition of two ribosomal RNAs being under two minutes.

In topology-independent superposition, ARTEMIS (mean TM-Score*_RNA_* 0.264, Figure 4A) surpasses US-align and Rclick (0.237 and 0.238, respectively). In this case, ARTEMIS also reports lower RMSD values (Figure 4B) and higher coverage values (Figure 4C). US-align is faster than ARTEMIS on short RNAs as evidenced by a nearly ten-time smaller median execution time value, while considerably slower on large RNAs as shown by a nearly eight-time larger mean value (Figure 4D).

We further evaluated the dependence of ARTEMIS execution time on the input size (Supplementary Figure S4). ARTEMIS demonstrates a better asymptotic time complexity compared to US-align, especially for topology-independent alignment, while Rclick shows a constant execution time for *M*> 200 due to the largest clique size threshold. Notably, US-align utilizes a fast mode starting at *M*= 1500 residues, while ARTEMIS runs in the accelerated mode starting at *M*= 500 residues.

Additionally to comparing mean TM-Score_RNA_ values, we evaluated the ARTEMIS performance in the worst case (Supplementary Figure S5, Supplementary Table S5). In comparison with US-align, ARTEMIS has shown a higher TM-Score_RNA_ for 75% of structures in sequentially-ordered alignments and for 76% of structures in topology-independent alignments. The respective values against the best competitor are 70% and 67%. In the worst case, TM-Score_RNA_ reported by ARTEMIS is lower than the best competitor by only 0.26, while in the best case, TM-Score_RNA_ reported by ARTEMIS is higher than the best competitor by 0.46. Only for 3 and 17 structures in sequentially-ordered and topology-independent alignments respectively the difference between the best competitor's TM-Score_RNA_ and the one reported by ARTEMIS exceeds 0.2, while the opposite difference exceeds 0.2 for 9 and 36 structures respectively. Overall, ARTEMIS significantly improves the worst case which places it closer to the optimal solution than the other tools.

### Analysis of tRNA-like structures

**Figure 5.**
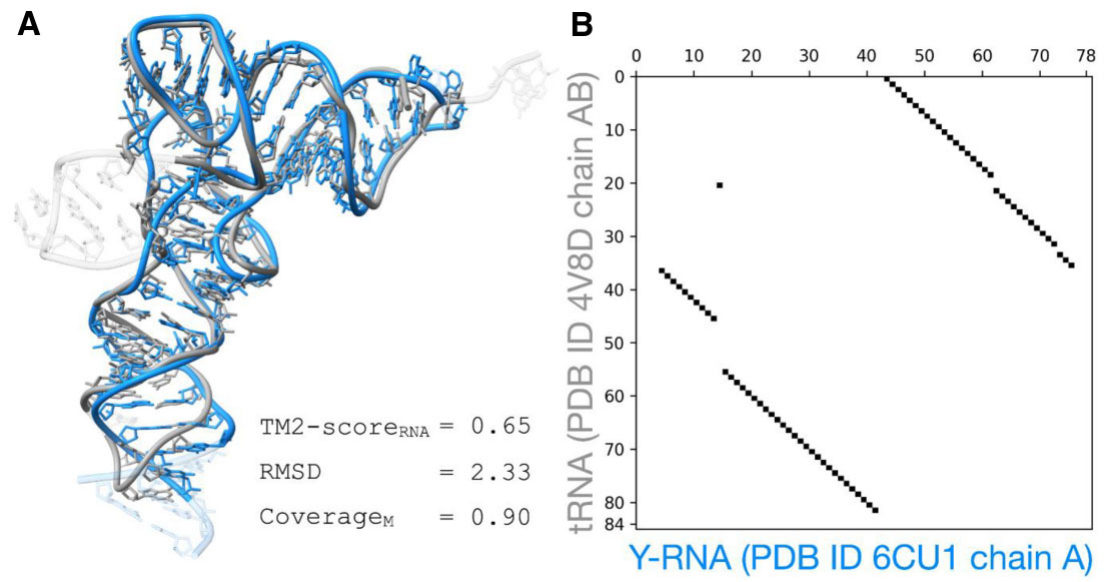
An illustration of a circularly permuted superposition between a bacterial Y RNA (PDB entry 6CU1, chain A) and a tRNA (PDB entry 4V8D, chain AB), identified by ARTEMIS. (**A**) 3D structure superposition. (**B**) Alignment plot.

Within the benchmark dataset, we identified 99 pairs of similar RNA 3D folds with sequence permutations (Supplementary Table S6). Among these pairs, 81 featured a bacterial Y RNA molecule (PDB entry 6CU1, chain A) (11) forming circularly permuted superpositions with various tRNAs (Figure 5). Subsequently, we manually collected a representative set of a tRNA molecule (28) and five tRNA-like structures (tmRNA fragment (29), selenocysteine tRNA (30), tRNA-like structure from Turnip Yellow Mosaic Virus (10), bacterial Y RNA (11), and tRNA-like structure from Brome Mosaic Virus (31)) and evaluated their pairwise topology-independent superpositions constructed by ARTEMIS, US-align, Rclick, and RNAhugs (Table 1, Supplementary Table S7).

**Table 1.** Performance of the topology-independent superposition tools on tRNA-like structures

Tool				ARTEMIS		US-align		Rclick		RNAhugs GEOS		RNAhugs GENS	
tRNA vs.	PDB id Chain 1ivsC	Length 75	Permutation	TM1 score	TM2 score	TM1 score	TM2 score	TM1 score	TM2 score	TM1 score	TM2 score	TM1 score	TM2 score
tmRNA fragment	2czjB	62	no	0.390	0.435	0.365	0.413	0.340	0.379	0.328	0.351	0.339	0.376
tRNA-Sec	3addC	88	no	0.453	0.485	0.461	0.494	0.445	0.477	0.446	0.473	0.446	0.473
TYMV tRNA-like	4p5jA	83	non-circular	0.450	0.468	0.464	0.481	0.407	0.416	0.430	0.440	0.421	0.430
Y RNA	6cu1A	79	circular	0.613	0.632	0.613	0.633	0.612	0.631	0.554	0.566	0.546	0.556
BMV tRNA-like	7samA	169	non-circular	0.261	0.372	0.210	0.300	0.273	0.401	0.244	0.378	0.196	0.291
**Mean**				0.433	0.478	0.423	0.464	0.415	0.461	0.400	0.442	0.390	0.425

**Figure 6.**
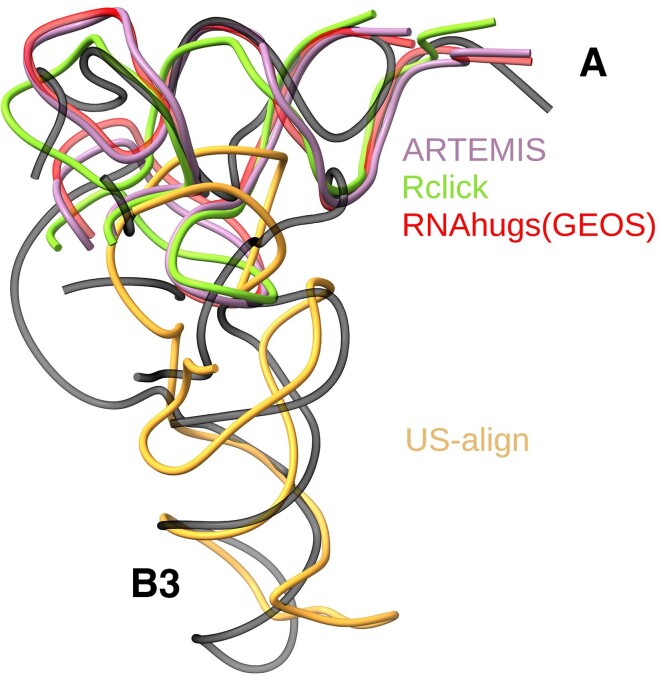
Topology-independent superposition of a tRNA (PDB entry 1IVS, chain C) and a BMV tRNA-like structure (PDB entry 7SAM, chain A, in black) conducted by ARTEMIS (in purple), US-align (in orange), Rclick (in green), and RNAhugs(GEOS) (in red). Unaligned structure fragments are hidden for clarity. Labels A and B3 denote the tRNA-mimicking fragments. The incorrect superposition by RNAhugs(GENS) is not shown.

ARTEMIS demonstrated superior *TM-Score_RNA_* values, although all tools performed very closely (Table 1). Interestingly, all tools generated almost identical superpositions for all structures except the tRNA-like structure from Brome Mosaic Virus (BMV, PDB entry 7SAM, chain A). The BMV structure includes two tRNA-mimicking fragments, with helical fragment A mimicking the tRNA acceptor stem and helical fragment B3 mimicking the tRNA anticodon stem (31). These two fragments cannot be superimposed on a tRNA structure simultaneously due to their extended arrangement. ARTEMIS, Rclick, and RNAhugs(GEOS) correctly superimposed fragment A onto the acceptor stem, while US-align correctly superimposed fragment B3 onto the anticodon stem (Figure 6). RNAhugs(GENS) reported an incorrect superposition.

Additionally, we used ARTEMIS in *-superonly* mode to assess conformational changes between different states of the tRNA-like structure from Brome Mosaic Virus (31) (Supplementary Table S8). This RNA was determined in three different states, one isolated state (PDB entry 7SAM, chain A), and two protein-bound states (PDB entries 7SC6 and 7SCQ, chain C). ARTEMIS reported TM-Score_RNA_ of 0.781 and RMSD of 3.26 Å between the protein-bound states. For the isolated state, ARTEMIS reported TM-Score_RNA_ of 0.687 and RMSD of 14.24 Å (against 7SC6) and TM-Score_RNA_ of 0.630 and RMSD of 14.07 Å (against 7SCQ). Thus, ARTEMIS allows to evaluate structural dynamics between different functional states of an RNA molecule.

### Analysis of the minor-groove/minor-groove helical packing motif

Among the 18 backbone-permuted structure pairs not involving the bacterial Y RNA, we observed seven pairs featuring the well-known minor-groove/minor-groove helical packing motif (32), often formed with widespread A-minor interactions (33) and GNRA-tetraloop/receptor motifs (34) (Supplementary Table S6).

**Figure 7.**
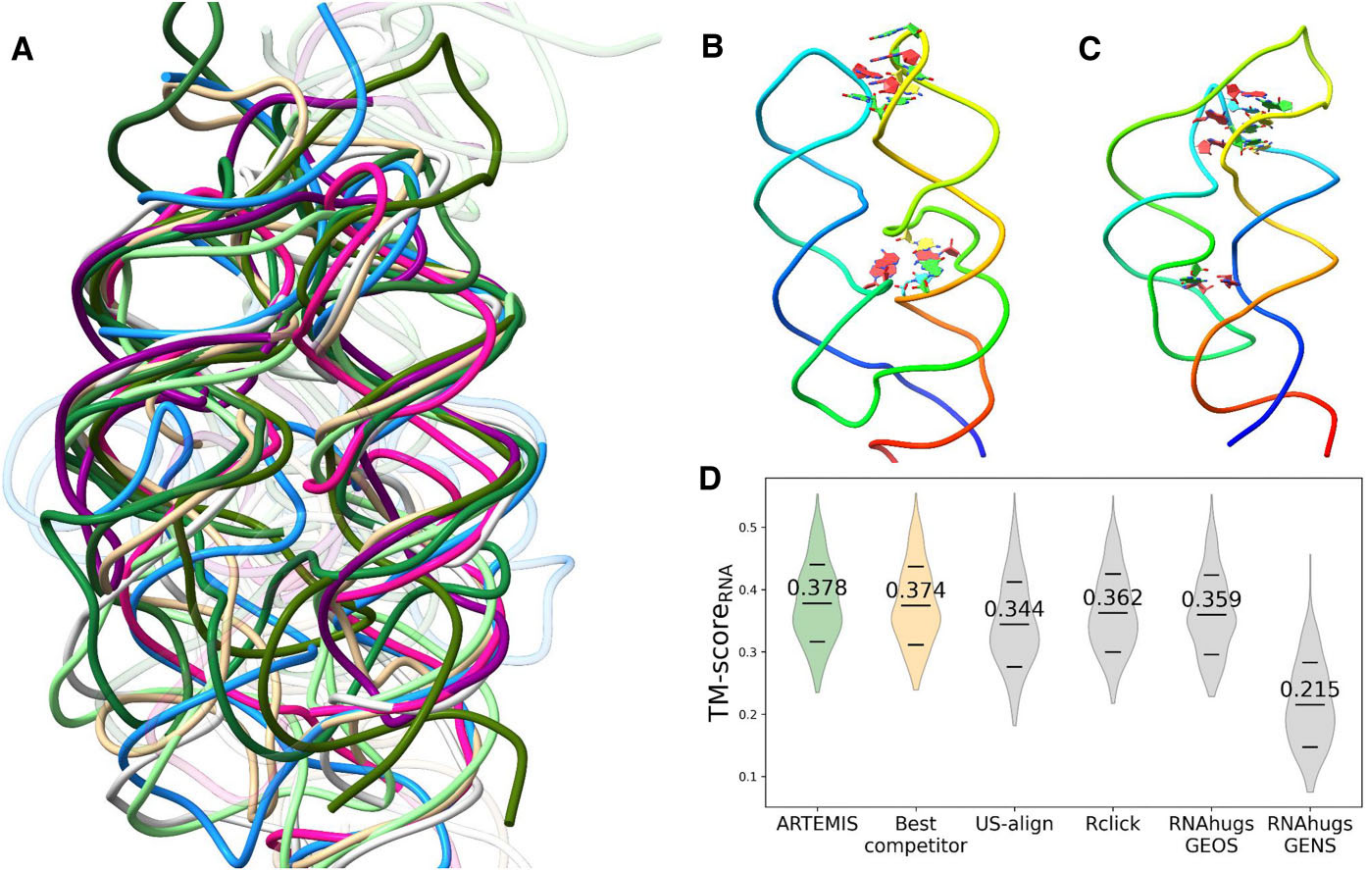
Minor-groove/minor-groove helical packing motif. (**A**) Topology-independent superposition constructed with ARTEMIS. (**B**) Archaeal SRP RNA (PDB entry 3NDB, chain M) forming a GNRA-tetraloop/receptor motif (top) and two A-minor interactions (bottom) and (**C**) THF riboswitch (PDB entry 6Q57, chain A) forming four A-minor interactions exhibit distinct backbone topology contexts. The chains are rainbow-colored, beginning with blue at the 5′-end and ending with red at the 3′-end. (**D**) Comparisons of topology-independent superpositions built by different tools. Mean ± standard deviation values are shown with dashes.

To analyze backbone variations of the helical packing motif we manually collected 16 representative RNA 3D structures featuring the motif. 15 structures were visually identified among 3D structures of non-coding RNA families from Rfam (35) and one synthetic RNA was found in the benchmark dataset (Table 2, Figure 7A). This set included various ribozymes and riboswitches with no obvious evolutionary relations, illustrating the motif's intrinsic presence in RNA structures. Remarkably, the helices packed similarly within completely different backbone topologies (Figure 7B, C). 11 of 16 motifs included A-minor interactions, five included GNRA-tetraloop/receptor interactions and one featured a D-loop/T-loop-like interaction (20). Benchmarks of the topology-independent superposition tools based on the selected set of 16 RNAs confirmed the superior performance of ARTEMIS (Figure 7D, Supplementary Figure S6, Supplementary Table S9).

**Table 2.** Representative RNA 3D structures featuring the minor-groove/minor-groove helical packing motif

#	PDB entry	RNA chain	Length	Rfam ID	Name	Helical packing motif interactions
1	2QUS	A	69	RF00008	Hammerhead ribozyme (type III)	GNRA-tetraloop(43–46)/receptor(18–26) A-minor (34–54-11, 35–53-12)
2	3D2G	A	77	RF00059	TPP riboswitch (THI element)	A-minor (44–9-37, 72–8-38)
3	6CC3	A	100	RF00080	yybP-ykoY manganese riboswitch	ribose-ribose (19–72) A-minor (10–61-37)
4	6UBU	B	67	RF00167	Purine riboswitch	A-minor (23–46–53, 33–37–61, 65–61–37, 66–38–60)
5	3DIG	X	174	RF00168	Lysine riboswitch	ribose-ribose (15–82) A-minor (81–14–78)
6	3L3C	P	154	RF00234	glmS glucosamine-6-phosphate activated ribozyme	ribose-ribose (81–88) A-minor (135–70–80)
7	3PDR	A	161	RF00380	M-box riboswitch (ykoK leader)	ribose-ribose (50–141)
8	5T83	A	90	RF00442	Guanidine-I riboswitch	A-minor (82–16–41, 83–15-42)
9	3IWN	A	93	RF01051	Cyclic di-GMP-I riboswitch	GNRA-tetraloop(22–25)/receptor(49–53,72–74) A-minor (39–12–35)
10	4FRN	A	102	RF01689	AdoCbl variant RNA	D-loop/T-loop-like (28–36, 57–61)
11	4ZNP	A	73	RF01750	ZMP/ZTP riboswitch	ribose-ribose (38–58) A-minor (39–69-57)
12	4P8Z	A	188	RF01807	GIR1 branching ribozyme	GNRA-tetraloop(158–161)/receptor(4–6, 183–185)
13	6Q57	A	89	RF01831	THF riboswitch	A-minor (8–34–44, 22–19–66, 23–18–67, 56–20–65)
14	4WFM	A	103	RF01854	Bacterial large signal recognition particle RNA	ribose-ribose (13–67) base-base (12–64)
15	3NDB	M	136	RF01857	Archaeal signal recognition particle RNA	GNRA-tetraloop(209–212)/receptor(165–166) A-minor (177–198-223, 176–194–224)
16	6DVK	H	95	Synthetic	mini tetraloop-tetraloop receptor construct	GNRA-tetraloop(46–49)/receptor(7–9, 85–87)

We then selected the helical packing motif from the THF riboswitch (Supplementary Figure S7A) as a representative instance and identified its matches in the benchmark dataset (Supplementary Table S10). We found 42 matches with *TM2-Score_RNA_* ≥ 0.45 belonging to 38 RNA structures of the dataset. This shows ARTEMIS’ unique ability to report sub-optimal matches which allows the identification of multiple instances of a query motif in a given RNA structure (Supplementary Figure S7).

### Analysis of non-coding RNA families

We used topology-independent alignments built by ARTEMIS for RNA structures of the benchmark dataset to analyze structural similarities within and between non-coding RNA families annotated in the Rfam database (35). The dataset included structures of 97 families, with 37 families represented with at least two structures. The mean TM-Score_RNA_ values reported for RNA structures of the same family, different families of the same clan, and from different clans are 0.61 (std = 0.18), 0.50 (std = 0.22), and 0.29 (std = 0.12) respectively (Supplementary Figure S8), which is in agreement with the 0.45 cut-off threshold suggested for similar folds in (2).

**Figure 8.**
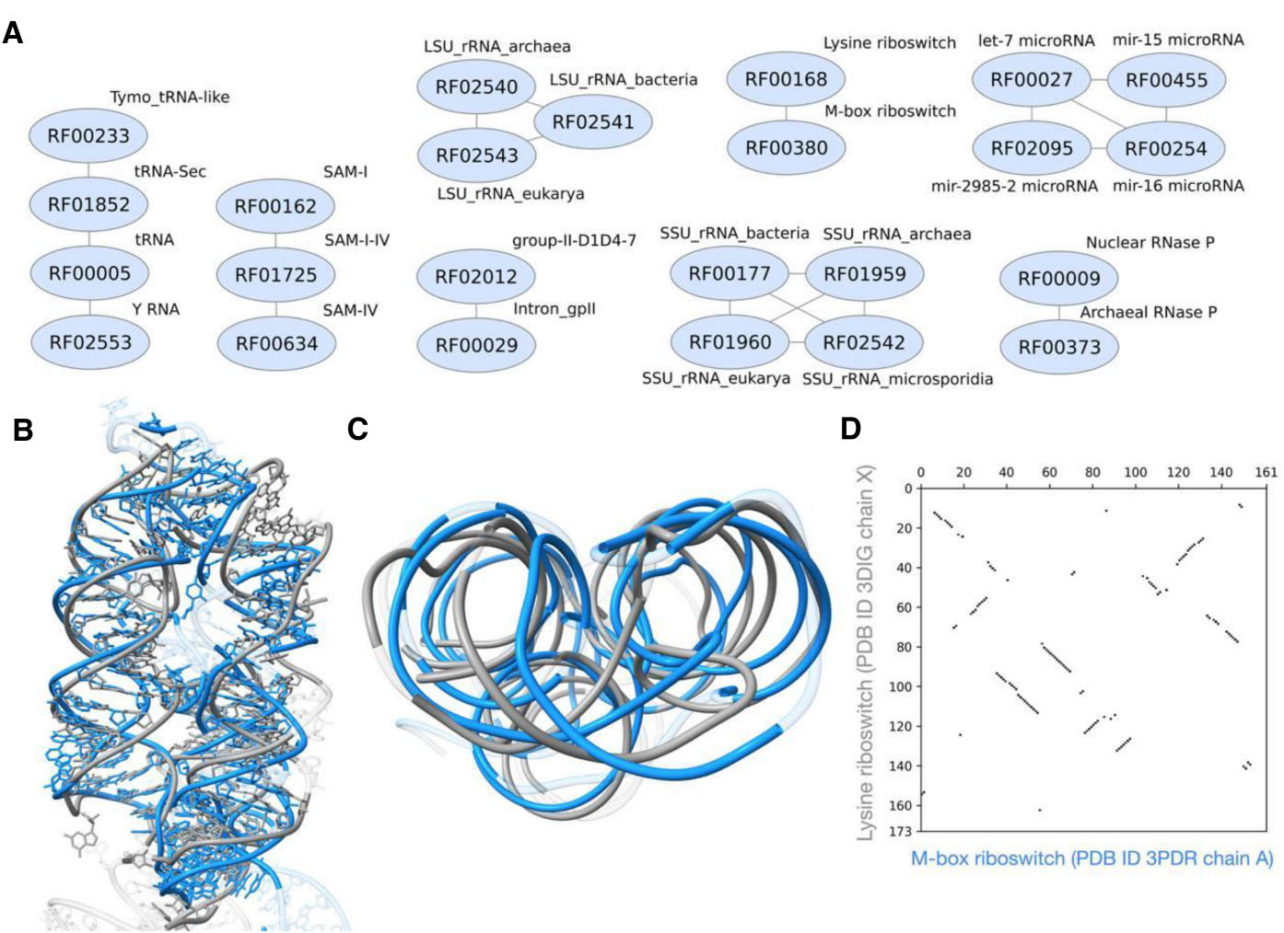
(**A**) A graph of non-coding RNA families sharing structural similarities, defined as having TM1-Score_RNA_ > 0.45 reported by ARTEMIS in topology-independent mode. (**B**) Side view, (**C**) Top view, and (**D**) 2D plot of topology-independent alignment built by ARTEMIS for Lysine riboswitch (PDB entry 3DIG, chain X) and M-box riboswitch (PDB entry 3PDR, chain A).

We further analyzed clusters of structurally similar families, whose structures were aligned by ARTEMIS with TM1-Score_RNA_ > 0.45 (Figure 8A). Among the eight clusters that we identified, only a single cluster represents a similarity that was not reported previously. This cluster comprises the Lysine riboswitch family (RF00168) and the M-box riboswitch family (RF00380). The structures of the two families have remotely similar 3D folds composed of three packed helices (Figure 8B, C). However, some helical regions are aligned in the reversed order, with major grooves of one riboswitch matching minor grooves of the other (reversed diagonals in Figure 8D). Therefore, this structural similarity is unlikely to point to an evolutionary relationship, but rather to shared physicochemical principles that dictate the packing of helical backbones.

## Discussion

In this work, we introduced ARTEMIS, a new method for RNA 3D structure superposition. Our benchmarks demonstrate that ARTEMIS significantly outperforms state-of-the-art methods in both sequentially-ordered and topology-independent (potentially sequence-permuted) superpositions. Yet, ARTEMIS relies on a simple heuristic of finding a single-residue match that has a near-zero RMSD in the optimal superposition. We believe this assumption is straightforward and stands out from the heuristics used in many other tools.

We utilized ARTEMIS to delve into the realm of backbone-permuted RNA structural similarities. The majority of such instances among the RNA 3D structures reported so far were tRNAs and tRNA-mimicking Y RNAs, alignable to each other with a circular permutation (Figure 5). Additionally, ARTEMIS successfully aligned tRNAs with viral tRNA-mimic structures, where the superpositions represent non-circular permutations (Table 1).

Through analysis of the RNA 3D structures with sequence permutations using ARTEMIS, we identified the minor-groove/minor-groove helical packing motif and showed that it is characteristic of multiple unrelated RNA molecules and can be formed within various backbone topology contexts (Figure 7). Based on our experience in RNA-Puzzles (36) and CASP15 (37,38), such long-range interactions involving not just the individual residues but entire secondary structure elements are not yet properly captured by any of the RNA 3D modeling programs. ARTEM and ARTEMIS can capture recurring interactions in RNA 3D structures involving multiple local structural motifs, which may hold a key to understanding the folding of large RNA molecules that rely on long-range interactions. Searches and comparisons can also include functional motifs such as protein and ligand binding sites, and ribozyme active sites.

Unlike other tools, ARTEMIS offers the capability for users to specify parts of the input structures to be used for superposition and separately designate which parts of the query coordinate file will be saved as the superimposed structure. This feature eliminates the need for manual editing of input coordinate files and proves useful e.g. for superimposing macromolecular complexes, including RNA-protein complexes, based on specified RNA chains, fragments, or even particular residues. Consequently, ARTEMIS can align multiple RNA chains simultaneously and its topology-independent mode can be used for inferring chain matchings if unknown. ARTEMIS is also suitable for the superposition of DNA molecules, including G-quadruplexes of different topological arrangements, although for G-quadruplexes, Rclick performs comparably to ARTEMIS (data not shown). Furthermore, ARTEMIS can recognize all types of modified residues (e.g. according to (39)) if the user adds their atomic representations to the config files (*artemis_1.json* and *artemis_2.json* files, see https://github.com/david-bogdan-r/ARTEMIS/tree/main/src/resrepr).

We anticipate that ARTEMIS will find utility in various studies related to 3D structures of RNA, DNA, and nucleic acid-containing complexes. It will prove particularly valuable for the comparative analysis of RNA 3D folds and motifs featuring sequence permutations. Our plans include extending ARTEM/ARTEMIS to accommodate protein structures and ligands, performing multiple structure/sequence alignments, and flexible 3D structure superpositions.

## Supplementary Material

gkae758_Supplemental_Files

## Data Availability

An implementation of the ARTEMIS tool and benchmarking data are available at https://github.com/david-bogdan-r/ARTEMIS (DOI: 10.5281/zenodo.13318963).
