# Supplementary material for: ARTEMIS: a method for topology-independent superposition of RNA 3D structures and structure-based sequence alignment
Source: Nucleic Acids Res. 2024 Sep 11;52(18):10850–61. doi: 10.1093/nar/gkae758 (PMC11472068; doi:10.1093/nar/gkae758)
Supplement: gkae758_Supplemental_Files [file gkae758_supplemental_files.zip › ARTEMIS_NAR_revised_supplementary.pdf]

SUPPLEMENTARY MATERIALS

ARTEMIS - a method for topology-independent superposition of RNA 3D structures and structure-based sequence alignment

Davyd R. Bohdan[1,*], Janusz M. Bujnicki[1], Eugene F. Baulin[1]

1. International Institute of Molecular and Cell Biology in Warsaw, Warsaw, Poland;

**corresponding author: dbohdan@iimcb.gov.pl, dav.bog.rom@gmail.com*

correspondence may also be sent to:

Janusz M. Bujnicki (janusz@iimcb.gov.pl), Eugene F. Baulin (efbaulin@gmail.com)

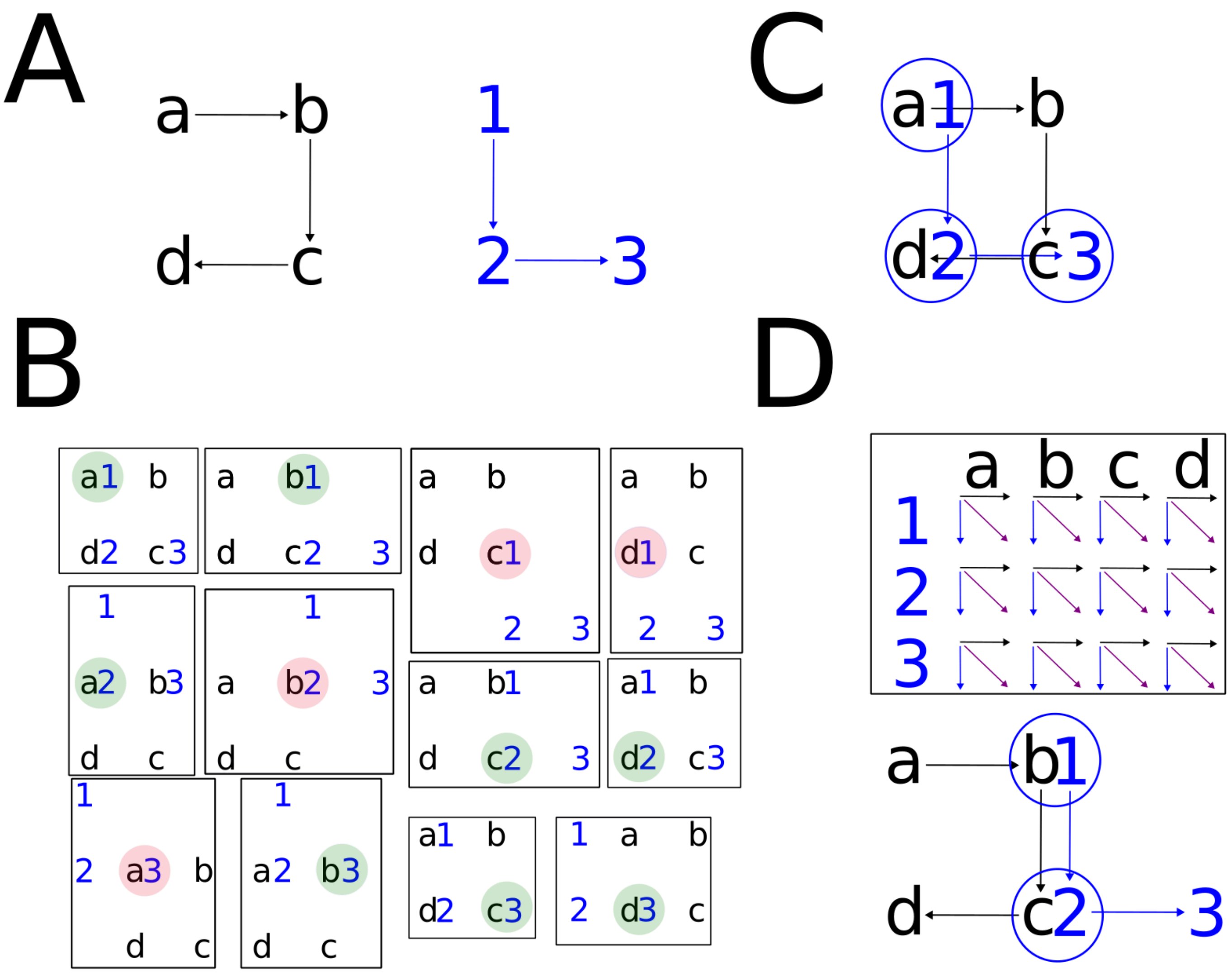

Supplementary Figure S1. An illustration of the ARTEMIS algorithm concept. (A). We have two input structures that are directed sequences of residues: reference structure abcd and query structure 123; (B) We superimpose the structures based on all possible single-residue matches: a-1, b-1, c-1, d-1, a-2, b-2, c-2, d-2, a-3, b-3, c-3, d-3. A subset of the largest hits (highlighted in green) is selected for the subsequent procedure, and the other hits (highlighted in red) are discarded; (C) The best topology-independent alignment is selected among the largest hits; (D) The largest hits undergo the Needleman–Wunsch procedure to identify the best sequentially-ordered alignment.

```
X[1..N]  = array of Reference RNA 3D structure residues
Y[1..M]  = array of Query     RNA 3D structure residues

X3[1..N] = 3-atom representations of X residues
Y3[1..M] = 3-atom representations of Y residues

X1[1..N] = C3' atom coordinates   of X residues
Y1[1..M] = C3' atom coordinates   of Y residues

MATCHRANGE1 = 3.5 Å # used in determining HIT
MATCHRANGE2 = 8   Å # used in determining ALIti

TOPLARGEST  = M
SHIFT2      = 3 Å

ALIARRAYsq  = [] # array of sequential alignments
ALIARRAYti  = [] # array of topology-independent alignments

HITARRAY    = []

for i in 1..N:
    for j in 1..M:
        rot, tran = KABSCH([(X3[i], Y3[j])])
        Y1' = Y1 * rot + tran

        # HIT - set of mutually closest residues
        HIT = [(r, q) | dist(X1[r], Y1'[q])
               == min(d(X1[r], Y1'[k]) for k in 1..M)
               == min(d(X1[k], Y1'[q]) for k in 1..N)
               < MATCHRANGE1]

        HITARRAY.append(HIT)

sort HITARRAY by descending HIT size

for HIT in HITARRAY[1..TOPLARGEST]:

    rot, tran = KABSCH([(X3[r], Y3[q]) for r, q in HIT])
    Y1' = Y1 * rot + tran

    ALIti = [(r, q) | dist(X1[r], Y1'[q])
             == min(d(X1[r], Y1'[k]) for k in 1..M)
             == min(d(X1[k], Y1'[q]) for k in 1..N)
             < MATCHRANGE2]

    SHIFT1   = max(dist(X1[r], Y1'[q]) for r, q in ALIti)
    SHIFT    = SHIFT1 + SHIFT2
    SCOREMAT = [[-dist(X1[r], Y1'[q]) + SHIFT for r in 1..N] for q in 1..M]

    ALIsq = NEEDLEMANWUNSCH(X, Y, SCOREMAT)

    ALIARRAYsq.append(ALIsq)
    ALIARRAYti.append(ALIti)

ALIsq = max(ALIARRAYsq by TM1scoreRNA + TM2scoreRNA)
ALIti = max(ALIARRAYti by TM1scoreRNA + TM2scoreRNA)
```

Supplementary Figure S2. ARTEMIS algorithm pseudocode.

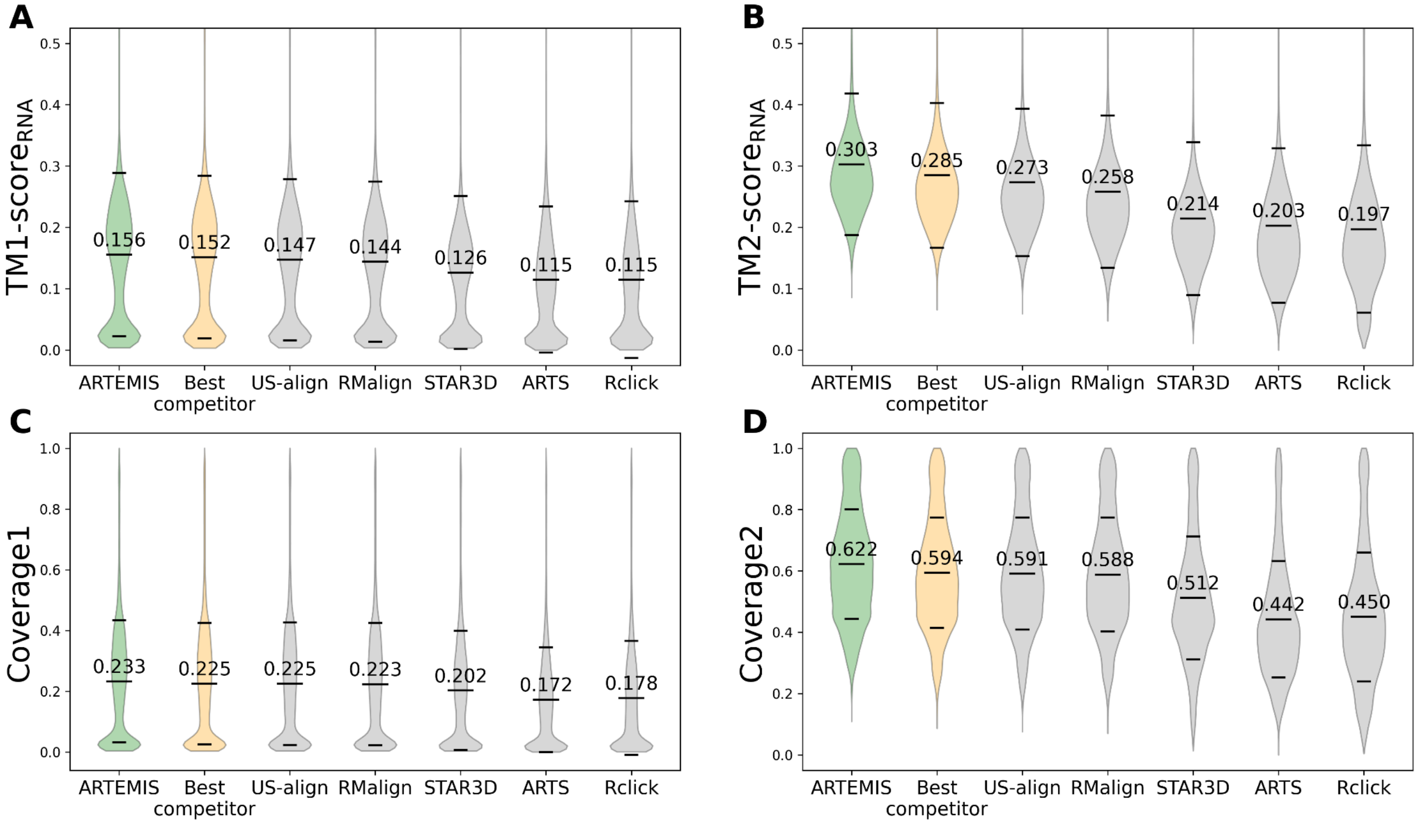

Supplementary Figure S3. Sequentially-ordered alignment benchmark results. Performance metrics include (A) TM1-score$_{RNA}$, (B) TM2-score$_{RNA}$, (C) coverage1, and (D) coverage2 values. ARTEMIS demonstrates superior performance. Mean +/- standard deviation values are specified with dashes. Panels B and D match the US-align benchmark results, see panels A and C of Figure 4 in [2] (10.1038/s41592-022-01585-1).

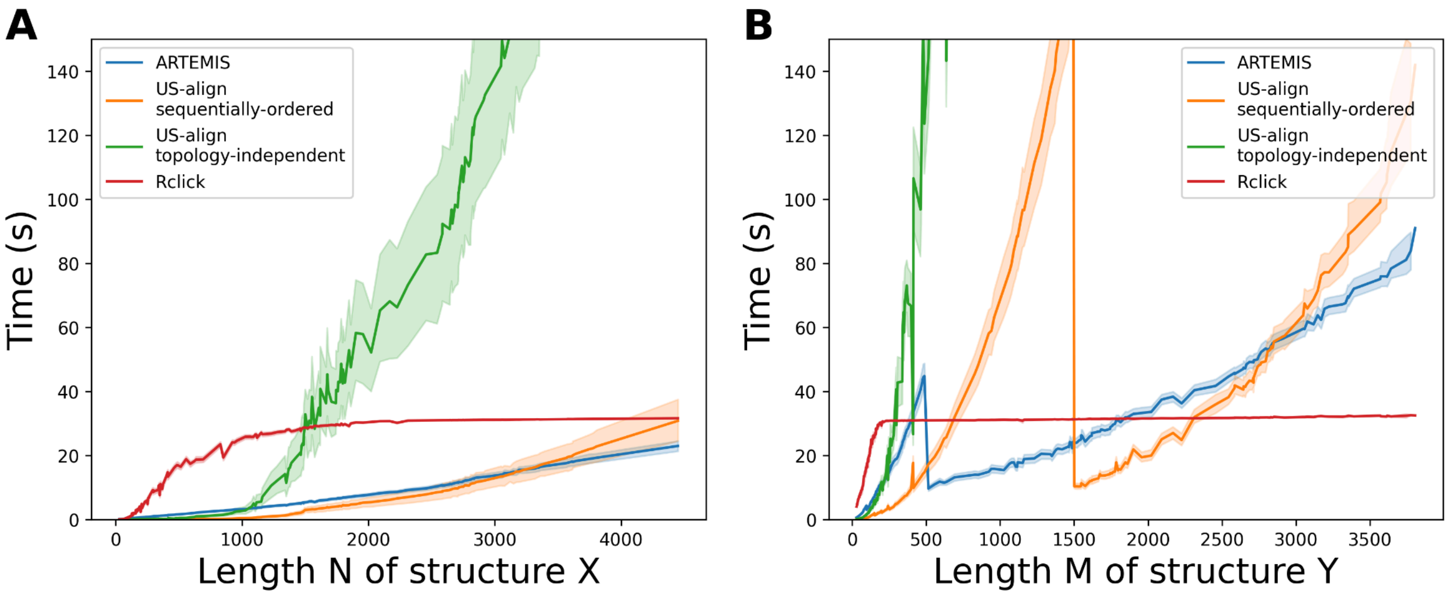

Supplementary Figure S4. The asymptotic time complexities of ARTEMIS, US-align, and Rclick calculated against (A) length N of larger structure X and (B) length M of smaller structure Y. ARTEMIS employs a fast mode starting at M = 500. US-align employs a fast mode starting at M = 1500 residues. Solid lines depict mean values and highlighted area shows 95% confidence interval estimates. Execution times of ARTEMIS and Rclick are identical between sequentially-ordered and topology-independent modes. The figure was prepared using Seaborn Python library (https://seaborn.pydata.org/generated/seaborn.lineplot.html).

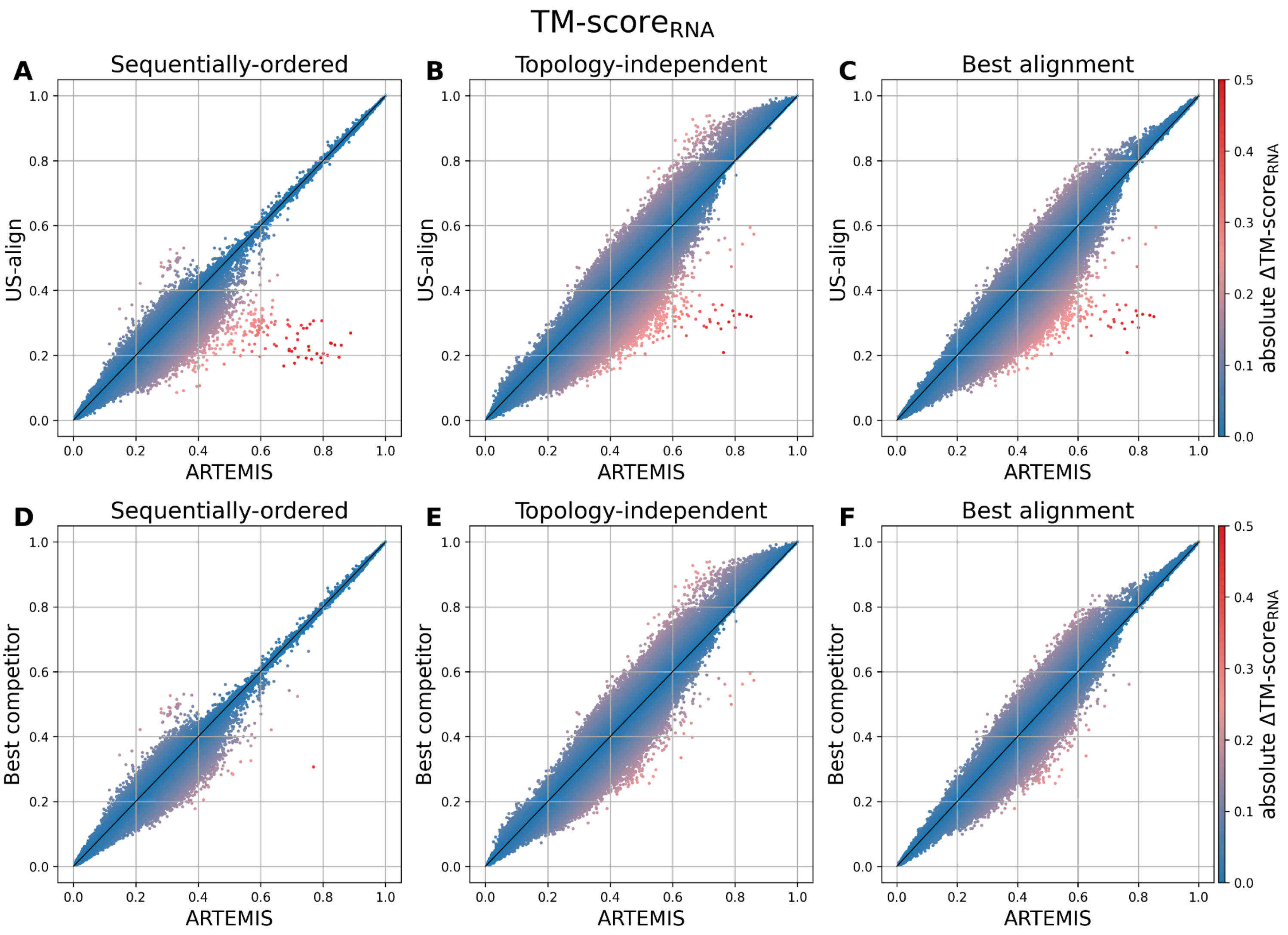

Supplementary Figure S5. TM-score$_{RNA}$ comparison between (A) ARTEMIS and US-align in sequentially-ordered and (B) topology-independent alignments, and (C) considering the best of the two alignments; (D) between ARTEMIS and the best competitor tool in sequentially-ordered and (E) topology-independent alignments, and (F) considering the best of the two alignments. Each dot represents a pair of RNA structures being aligned.

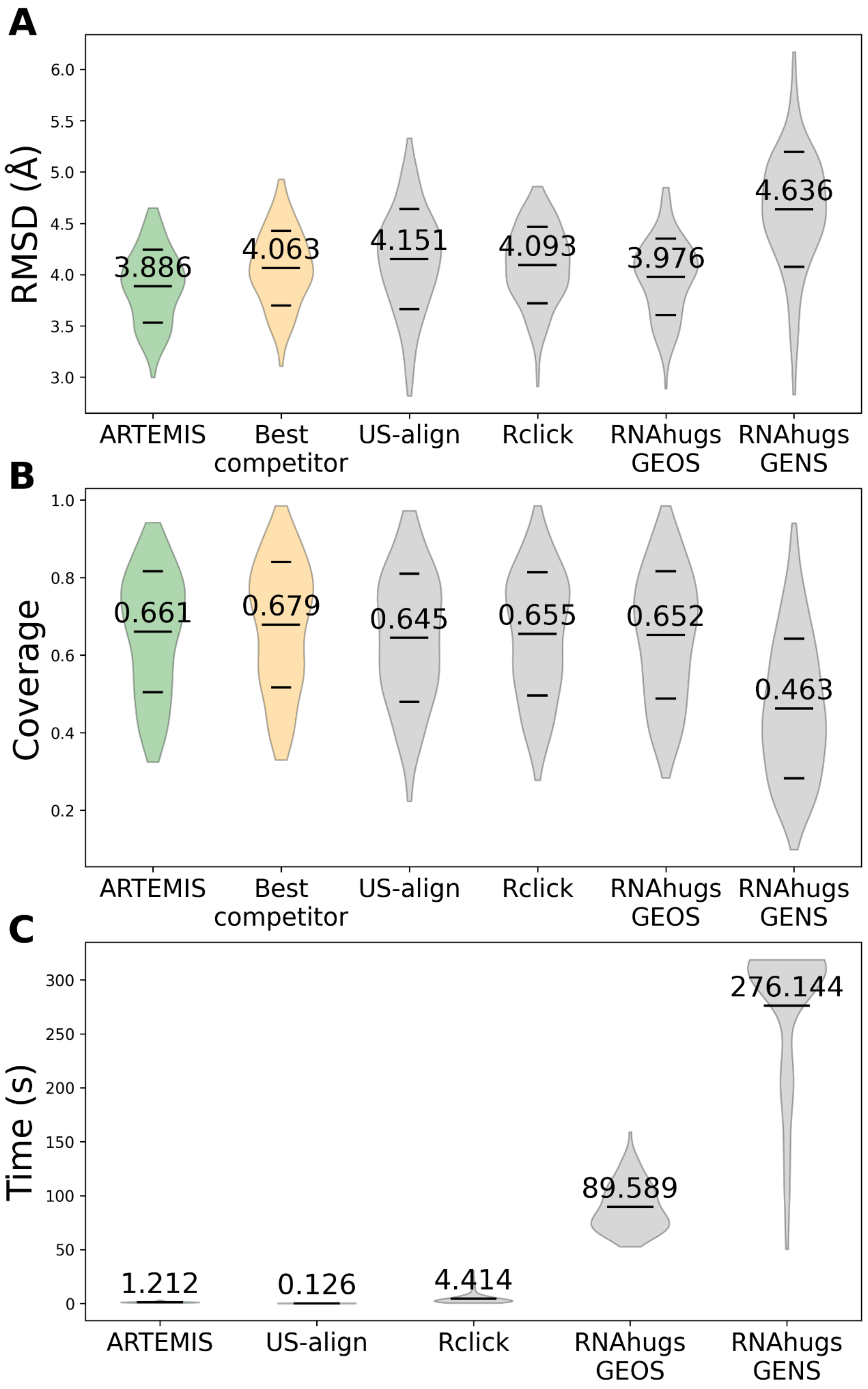

Supplementary Figure S6. Topology-independent superposition comparisons for RNAs featuring the minor-groove/minor-groove helical packing motif. Performance was measured by (A) RMSD, (B) coverage, and (C) execution time. ARTEMIS demonstrates superior performance compared to existing tools, as evidenced by lower RMSD values and higher coverage values.

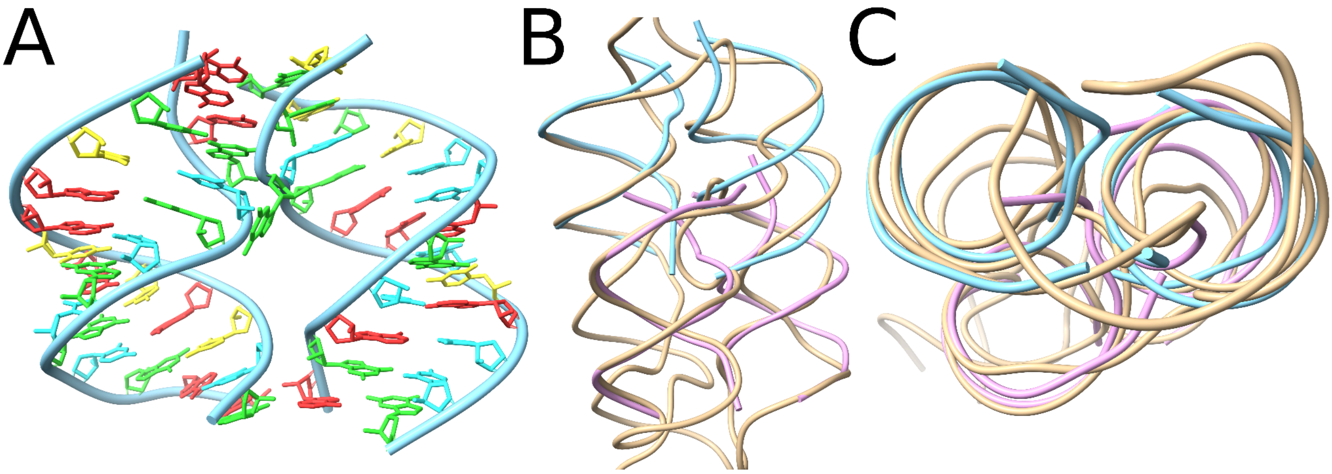

Supplementary Figure S7. (A) Representative minor-groove/minor-groove helical packing motif from a THF riboswitch (PDB entry 6Q57, chain A, residues 8-19, 22-34, 44-55, 66-78) and (B) a side view and (C) a top view of its two matches with TM2-Score$_{RNA}$ ≥ 0.45 (in blue and pink) in an M-box riboswitch (in beige, PDB entry 3PDR, chain A).

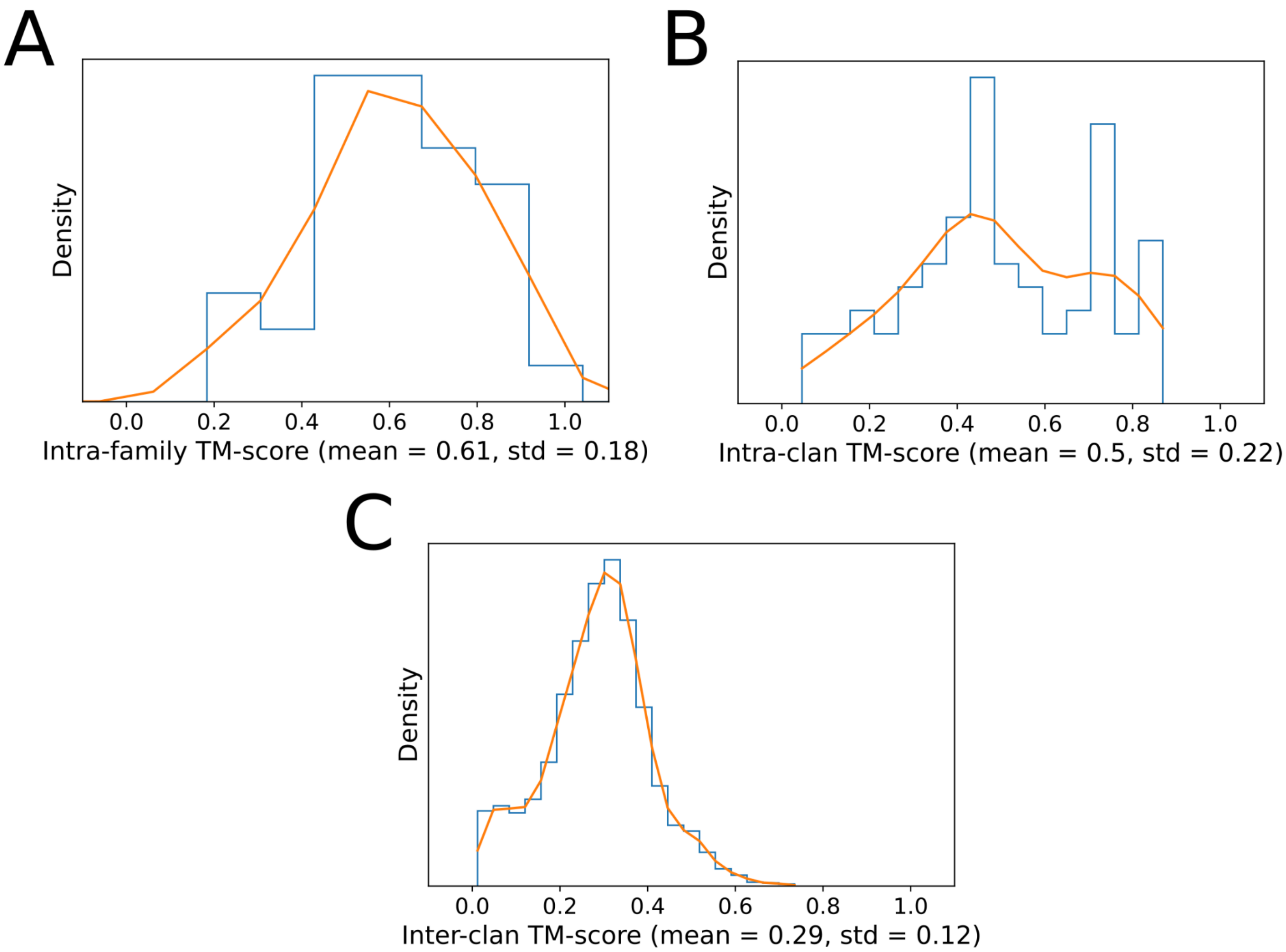

Supplementary Figure S8. The distributions of TM-scores reported by ARTEMIS for pairwise topology-independent alignments of RNA structures from the benchmark dataset. (A) Aligning structures of the same Rfam family. (B) Aligning structures of different Rfam families of the same Rfam clan. (C) Aligning structures of different Rfam clans.

Supplementary Table S1. The list of 500 RNA structure pairs used in the preliminary experiments for the pseudo atom selection. Structure identifiers are built by concatenating PDB entries and chain identifiers, separated with dots.

1rnk.A 1hvu.C, 2xdb.G 4ato.G, 4pcj.A 6vu1.A, 3ovb.D 2zh3.B, 6dcb.B 3ovb.D, 2fdt.A 1e95.A, 4pdb.I 4pcj.A, 4pdb.I 1i6u.C, 4pdb.I 6d12.C, 4kr6.C 1et4.A, 5xut.B 6dcb.B, 5nfv.B 5v3f.A, 5nfv.B 5xut.B, 2kx8.A 5voe.A, 5m0h.A 4pcj.A, 3iab.R 6vu1.A, 1xjr.A 4pdb.I, 2nue.C 3p22.A, 5ztm.C 2lwk.A, 2m8k.A 2kpv.A, 1rmn.A 1ekz.B, 6qn3.A 1z2j.A, 6r47.A 5k7d.A, 6r47.A 6ufj.A, 6e9f.B 6dcb.B, 2qwy.A 4rmo.B, 4enc.A 5kh8.A, 2n4l.A 4oog.D, 2n4l.A 3p22.A, 2n4l.A 5ztm.C, 6lax.A 6sdw.B, 4k27.U 4pcj.A, 4v2s.Q 4pjo.4, 5zal.C 6mxq.A, 5zal.C 2n4l.A, 5u0a.K 4x4v.B, 5u0a.K 2zh3.B, 3izz.A 6c65.A, 4m4o.B 5ztm.C, 5u0a.K 5dqk.A, 5h9f.L 5y85.B, 4u7u.L 3zp8.A, 5btp.A 1zho.B, 2czj.B 1p6v.B, 5btp.A 5ztm.C, 5ylz.E 6ff4.Z, 2czj.B 4pkd.V, 2czj.B 2nre.F, 3icq.D 4m4o.B, 6rfl.U 2fk6.R, 5b63.D 2fk6.R, 5b63.D 3icq.D, 5b63.D 6rfl.U, 1ser.T 2fk6.R, 2nc1.A 2m8k.A, 3ski.B 6mxq.A, 6gaw.BB 2fk6.R, 6gaw.BB 3j7y.B, 1h4s.T 3icq.D, 1h4s.T 6rfl.U, 6p2h.A 6d3p.A, 6p2h.A 4fe5.B, 3eph.E 2fk6.R, 5ob3.A 1dk1.B, 3hay.E 3lwr.D, 3eph.E 2czj.B, 3ivn.A 6p2h.A, 1kxk.A 4pcj.A, 1kxk.A 5m0h.A, 2l3j.B 2n6s.A, 5kpy.A 2ke6.A, 2l3j.B 5zal.C, 2du3.D 6rfl.U, 2du3.D 5b63.D, 3la5.A 3ivn.A, 4by9.A 3nmu.D, 6lxd.D 2nue.C, 6lxd.D 4m4o.B, 5wwr.C 1h4s.T, 5wwr.C 3eph.E, 5czz.B 2nue.C, 3wc1.P 2n4l.A, 6cae.1y 2n4l.A, 4znp.A 5btp.A, 6cae.1y 6rfl.U, 5e6m.C 6rfl.U, 6cae.1y 1h4s.T, 5e6m.C 1h4s.T, 3wc1.P 3eph.E, 6cae.1y 3eph.E, 3wc1.P 6gaz.AV, 5e6m.C 2du3.D, 3wc1.P 2du3.D, 6cae.1y 2du3.D, 1j1u.B 2fk6.R, 2j28.8 4m4o.B, 1j1u.B 3icq.D, 4yye.C 6rfl.U, 1j1u.B 6rfl.U, 2d6f.F 6rfl.U, 2der.C 6rfl.U, 4yye.C 5b63.D, 3j7a.7 5b63.D, 2j28.8 6cae.1y, 2der.C 3wc1.P, 1j1u.B 2dlc.Y, 3j7a.7 6cae.1y, 6tbv.PTR1 4x4v.B, 2csx.C 4x4v.B, 2zue.B 2zh3.B, 2csx.C 2zh3.B, 1gax.C 2zh3.B, 6tbv.PTR1 2zh3.B, 2csx.C 4x4p.B, 1gax.C 2nue.C, 4v7l.AY 2nue.C, 6tb3.n 2nue.C, 2zue.B 2fk6.R, 6tb3.n 2fk6.R, 6tb3.n 5zal.C, 2zue.B 3icq.D, 6tbv.PTR1 3icq.D, 6tb3.n 3icq.D, 4v7l.AY 6rfl.U, 2csx.C 6rfl.U, 1gax.C 5b63.D, 6tb3.n 5b63.D, 6ip5.zu 5b63.D, 3wqy.C 5b63.D, 1il2.C 3eph.E, 2csx.C 3eph.E, 3wqy.C 3eph.E, 6ip5.zu 3j7a.7, 4lck.B 1j1u.B, 6tbv.PTR1 2der.C, 6tb3.n 2der.C, 1g59.B 3akz.E, 6tb3.n 1j1u.B, 6ip5.zu 4v7l.AY, 4v9i.AY 6tbv.PTR1, 3wqy.C 2csx.C, 6t7t.6 2zh3.B, 4wj4.B 2nue.C, 4v5g.AY 2nue.C, 2iy5.T 6mxq.A, 6r5q.2 2fk6.R, 3j46.p 2fk6.R, 6t7t.6 2fk6.R, 1qf6.B 2lc8.A, 3j46.p 5zal.C, 5el6.3K 3icq.D, 6r5q.2 3icq.D, 4rdx.C 6rfl.U, 6t7t.6 6rfl.U, 6r5q.2 6rfl.U, 6r87.B 6rfl.U, 4wj4.B 5b63.D, 6r87.B 5b63.D, 6t4q.6 5b63.D, 4wj4.B 1h4s.T, 4wj4.B 3eph.E, 4wj4.B 2du3.D, 6r5q.2 2du3.D, 5el6.3K 2zni.C, 6r5q.2 3wc1.P, 6t7t.6 3wc1.P, 4v6u.A0 3wc1.P, 6q9a.7 1j1u.B, 6t4q.6 2der.C, 6r5q.2 1j1u.B, 4v6u.A0 6tbv.PTR1, 6r87.B 6tb3.n, 4wj4.B 1il2.C, 6r5q.2 6tb3.n, 6t4q.6 6tbv.PTR1, 6t7t.6 4v9i.AY, 6q9a.7 6tbv.PTR1, 6r87.B 4v9i.AY, 4v6u.A0 4v9i.AY, 6r5q.2 4v9i.AY, 3j46.p 6q9a.7, 4v6u.A0 6q9a.7, 6t7t.6 6t4q.6, 6r87.B 6r5q.2, 6t7t.6 6az1.2, 6az1.2 6t4q.6, 6t7t.6 6r5q.2, 4v5g.AY 4v5l.AY, 6q9a.7 6t4q.6, 6r87.B 6t7t.6, 5lzs.ii 4v5l.AY, 3j46.p 6t4q.6, 6q9a.7 6r87.B, 4v6u.A0 6r5q.2, 5j8b.x 2zh3.B, 6svs.A 2fk6.R, 5j8b.x 2fk6.R, 6i0y.V 2fk6.R, 5o2r.x 2fk6.R, 6svs.A 5zal.C, 5j8b.x 6rfl.U, 6q97.7 6rfl.U, 6i0y.V 5b63.D, 6ufm.A 5b63.D, 5j8b.x 5b63.D, 6ugg.A 1h4s.T, 5j8b.x 2du3.D, 4v6u.A1 6cae.1y, 6ugg.A 3wc1.P, 5j8b.x 3wc1.P, 6i0y.V 3wc1.P, 6ugg.A 2j28.8, 6ufm.A 1j1u.B, 3a2k.C 4yye.C, 6ufm.A 2der.C, 6i0y.V 1j1u.B, 5o2r.x 2der.C, 6v3a.v 3j7a.7, 5j8b.x 2der.C, 5j8b.x 4v7l.AY, 6ugg.A 4v7l.AY, 3a2k.C 1il2.C, 6v3a.v 6t4q.7, 6i0y.V 6tbv.PTR1, 5j8b.x 4v9i.AY, 6i0y.V 6tb3.n, 6ufm.A 6tbv.PTR1, 4v6u.A1 6t4q.7, 5o2r.x 6tb3.n, 4v6u.A1 6ek0.S6, 6i0y.V 5el6.3K, 5o2r.x 6az1.2, 6i0y.V 6q9a.7, 6ufm.A 6t4q.6, 6i0y.V 3j46.p, 5o2r.x 6t7t.6, 5j8b.x 6q9a.7, 3a2k.C 4wj4.B, 6i0y.V 4v6u.A0, 5o2r.x 3j46.p, 5o2r.x 6t4q.6, 6ugg.A 4rdx.C, 5j8b.x 6r5q.2, 6ufm.A 6t7t.6, 6i0y.V 6t4q.6, 6i0y.V 6r5q.2, 4v6u.A1 5el6.3K, 5o2r.x 4v6u.A0, 6i0y.V 6r87.B, 6ufm.A 6r5q.2, 5o2r.x 6q9a.7, 6i0y.V 6t7t.6, 5o2r.x 5j8b.x, 6i0y.V 5j8b.x, 4v6u.A1 6v3a.v, 5o2r.x 6ufm.A, 6ufm.A 5j8b.x, 3amt.B 1p6v.B, 6v5b.D 2fk6.R, 3amt.B 2n4l.A, 6v5b.D 5zal.C, 5vpp.QV 6rfl.U, 3amt.B 5b63.D, 5vpp.QV 5b63.D, 5vpp.QV 1h4s.T, 6v5b.D 6lxd.D, 4v8n.AW 3wc1.P, 6rja.D 5czz.B, 5vpp.QV 6t4q.7, 5vpp.QV 6v3a.v, 6cu1.A 4znp.A, 6ah3.T 6rfl.U, 6ah3.T 1h4s.T, 4yaz.A 4m4o.B, 6b14.R 4v4p.B1, 6b14.R 6svs.A, 2zzm.B 3j7y.B, 1h3e.B 4v6u.A0, 4v8d.AB 6tbv.PTR1, 4v8d.AB 4v6u.A0, 4v8d.AB 6t4q.6, 4mgn.A 6b14.R, 5aox.F 2n4l.A, 5aox.F 5zal.C, 5aox.F 2qus.A, 4v8b.AB 6cu1.A, 4lvw.A 4rmo.B, 5t83.A 5y58.X, 5t83.A 5m0h.A, 5t83.A 6mxq.A, 5t83.A 4k27.U, 5t83.A 2mqt.A, 5t83.A 2qus.A, 5t83.A 2n6w.A, 5t83.A 5ob3.A, 3w1k.F 2nue.C, 1m5o.B 5zal.C, 3adb.C 1ser.T, 3cul.D 2mqt.A, 5x2h.B 5dqk.A, 3iwn.A 6lxd.D, 3iwn.A 5t83.A, 6dvk.H 2kx8.A, 5ml7.A 1u63.B, 6dvk.H 5zal.C, 6dvk.H 2l3j.B, 5lzd.y 6v3a.v, 6dvk.H 6v5b.D, 6jxm.B 2n4l.A, 4oqu.A 4jf2.A, 6jxm.B 4jf2.A, 6az3.5 5aox.F, 3w3s.B 2nre.F, 4rzd.A 5zal.C, 3w3s.B 5zal.C, 3w3s.B 3eph.E, 3w3s.B 3w1k.F, 3suh.X 4pkd.V, 3suh.X 4m4o.B, 6dmc.A 2qus.A, 6dmc.A 2j28.8, 3suh.X 4y1j.A, 6mj0.A 6jxm.B, 4lck.C 6vu1.A, 4frn.A 4oji.A, 2xxa.F 4m4o.B, 2xxa.F 2n6w.A, 4lck.C 5ob3.A, 2xxa.F 6v5b.D, 5nco.1 6b14.R, 3jb9.C 2l3j.B, 3jb9.C 6v5b.D, 6dlr.A 5nco.1, 3f2q.X 4qjd.B, 3f2q.X 6mwn.A, 2nbx.A 5zal.C, 2nbx.A 2l3j.B, 2nbx.A 6lxd.D, 2nbx.A 6v5b.D, 6ck5.A 6dlr.A, 1foq.A 2nue.C, 1foq.A 5ob3.A, 1foq.A 2l3j.B, 2lkr.A 2nue.C, 6ol3.C 2n6w.A, 6ol3.C 1kxk.A, 6ol3.C 2zni.C, 6ol3.C 6v5b.D, 6ol3.C 4mgn.A, 6ol3.C 1m5o.B, 6ol3.C 6jxm.B, 6ol3.C 6dmc.A, 6ol3.C 5nco.1, 5vt0.R 1kxk.A, 5vt0.R 6lxd.D, 6ahd.B 5zal.C, 6ahd.B 2l3j.B, 6ahd.B 6svs.A, 6ahd.B 6v5b.D, 6ahd.B 6jxm.B, 6v3a.B 4y1m.B, 6ahd.B 2nbx.A, 5xy3.3 5u3g.B, 4qln.A 6mwn.A, 6xyw.3 5nco.1, 6xyw.3 6dlr.A, 5o60.B 6v3a.B, 3j79.B 6s0x.B, 5o60.B 5myj.BB, 6rm3.L70 5xxb.3, 3jcs.8 1s9s.A, 3jcs.8 4y1m.B, 4v8m.BD 5xy3.3, 3jcs.8 5xxb.3, 4v8m.BD 5xxb.3, 3jcs.8 6rm3.L70, 4ybb.DB 2hgh.B, 4ybb.DB 5myj.BB, 4v8p.B3 5xy3.3, 4v6w.A7 5xy3.3, 4v7e.Ab 5xxb.3, 4v8p.B3 3j79.B, 4v8p.B3 5xxb.3, 4v6w.A7 3j79.B, 4v8p.B3 3jcs.8, 4v6w.A7 3jcs.8, 4v6w.A7 4v8m.BD, 4v6w.A7 4v8p.B3, 4v6w.A7 4v7e.Ab, 4v7e.Ab 4v8p.B3, 5mmi.B 5myj.BB, 5mmi.B 6s0x.B, 6t4q.C4 5xy3.3, 6t4q.C4 3j79.B, 6t4q.C4 6rm3.L70, 4v6x.A7 5xxb.3, 4v6x.A7 6rm3.L70, 4v6x.A7 3jcs.8, 4v6x.A7 4v8m.BD, 6t4q.C4 4v8p.B3, 4v6x.A7 4v8p.B3, 5mmi.B 4ybb.DB, 4v6x.A7 6t4q.C4, 5dm6.Y 5t5a.A, 1vq8.9 5nco.1, 1vq8.9 4y1m.B, 1vq8.9 5myj.BB, 1vq8.9 3j79.B, 1vq8.9 5xxb.3, 1vq8.9 6rm3.L70, 1vq8.9 6t4q.C4, 1vq8.9 4v6x.A7, 5dm6.Y 5mmi.B, 5zlu.X 5dqk.A, 5zlu.X 1un6.E, 5zlu.X 4y1m.B, 5zlu.X 5myj.BB, 5zlu.X 4ybb.DB, 5zlu.X 5mmi.B, 4v6u.B3 5t5a.A, 3v7e.C 5ob3.A, 3v7e.C 4jf2.A, 3v7e.C 3owi.B, 4v6u.B3 5xy3.3, 3v7e.C 5o60.B, 3v7e.C 3j3v.B, 3v7e.C 4v8m.BD, 3v7e.C 4v8p.B3, 3v7e.C 6t4q.C4, 6vvj.A 6vu1.A, 6vvj.A 6mxq.A, 6vvj.A 2n4l.A, 6vvj.A 5zal.C, 6vvj.A 2l3j.B, 6vvj.A 6lxd.D, 6vvj.A 6v5b.D, 6vvj.A 1foq.A, 4uyk.R 1kxk.A, 4uyk.R 2l3j.B, 4uyk.R 6v5b.D, 4uyk.R 2xxa.F, 4uyk.R 3jb9.C, 4uyk.R 1foq.A, 4uyk.R 6ol3.C, 4v8m.BH 1kxk.A, 2il9.A 6v5b.D, 2il9.A 2xxa.F, 2il9.A 6dlr.A, 2il9.A 6ck5.A, 2il9.A 4qln.A, 2il9.A 6xyw.3, 3ndb.M 2n4l.A, 3ndb.M 2xxa.F, 3ndb.M 6ol3.C, 3l3c.S 3cul.D, 3l3c.S 6dmc.A, 6jdv.B 6mxq.A, 6jdv.B 4k27.U, 6jdv.B 5zal.C, 6jdv.B 2n6w.A, 6jdv.B 2xxa.F, 1nbs.B 3ivn.A, 1nbs.B 6dmc.A, 6pom.A 6pmo.A, 6pom.A 5ob3.A, 2n1q.A 2l3j.B, 6pom.A 6ol3.C, 3pdr.A 3suh.X, 5wlc.L2 1zho.B, 3dil.A 4yaz.A, 3j5s.A 6ol3.C, 6az3.3 1m5o.B, 6az3.4 4ybb.DB, 6az3.4 5dm6.Y, 5v3i.A 6s0x.B, 5v3i.A 5xy3.3, 5v3i.A 6xyw.3, 5v3i.A 3j79.B, 5v3i.A 5o60.B, 5v3i.A 5xxb.3, 5v3i.A 3jcs.8, 5v3i.A 4v8m.BD, 5v3i.A 4ybb.DB, 5v3i.A 4v6w.A7, 5v3i.A 4v6x.A7, 5v3i.A 5dm6.Y, 4gma.Z 5k7d.A, 4gma.Z 6r47.A, 1u6b.B 5myj.BB, 4d5n.X 4v8p.B3, 4d5n.X 4v7e.Ab, 4c4q.N 5zal.C, 3j16.J 5a8l.B, 4c4q.N 6lxd.D, 4c4q.N 2xxa.F, 2rkj.C 4kqy.A, 5flx.z 5vt0.R, 6sxo.L5 2j28.8, 6sxo.L5 2xxa.F, 6sxo.L5 5nco.1

Supplementary Table S2. Technical details of running the standalone versions of superposition tools.

<table>
<tr><th>Tool</th><th colspan="2">Description</th></tr>
<tr><td rowspan="4">ARTEMIS</td><td>Downloaded from</td><td>https://github.com/david-bogdan-r/ARTEMIS</td></tr>
<tr><td>Installation details</td><td>version 1.5</td></tr>
<tr><td>Running command for topology-independent mode</td><td>python3 artemis.py r=inputPDB1 q=inputPDB2 -p</td></tr>
<tr><td>Running command for minor-groove/minor-groove helical packing motif search</td><td>python artemis.py q=MGMG_6q57.pdb -p saveto=MGMG r=pdb637/*.pdb addhits=0.3 > MGMG_637_TM30.tsv</td></tr>
<tr><td rowspan="3">Rclick</td><td>Downloaded from</td><td>https://mspc.bii.a-star.edu.sg/minhn/download.html</td></tr>
<tr><td>Installation details</td><td>tar.gz archive dated 22 November 2012;
Changed the typeAtom parameter from CA to C3′ in the Parameters.inp file</td></tr>
<tr><td>Running command</td><td>./click inputPDB2 inputPDB1</td></tr>
<tr><td rowspan="4">RNAhugs</td><td>Downloaded from</td><td>https://github.com/RNApolis/rnahugs</td></tr>
<tr><td>Installation details</td><td>commit a678cb4 on Dec 18, 2023</td></tr>
<tr><td>Command for GEOS mode</td><td>java -jar target/rna-hugs-1.0-jar-with-dependencies.jar --reference inputPDB1 --model inputPDB2 --method geometric</td></tr>
<tr><td>Command for GENS mode</td><td>java -jar target/rna-hugs-1.0-jar-with-dependencies.jar --reference inputPDB1 --model inputPDB2 --method genetic</td></tr>
<tr><td rowspan="4">US-align</td><td>Downloaded from</td><td>https://github.com/pylelab/USalign</td></tr>
<tr><td>Installation details</td><td>version 20220924 (as the topology-independent mode is not implemented in the original version 20220227)</td></tr>
<tr><td>Command for topology-independent mode</td><td>./USalign inputPDB2 inputPDB1 -mm 5</td></tr>
<tr><td>Command for topology-independent mode without re-superimposing input structures</td><td>./USalign superPDB2 superPDB1 -mm 5 -se</td></tr>
</table>

Supplementary Table S3. Student's paired t-test and Wilcoxon signed-rank test p-values calculated for each pair of the superposition tools based on the sets of TM-score$_{RNA}$ values, as measured on the dataset of 637 RNA chains in building sequentially-ordered alignments. Zeros correspond to values < 1e-303.

**Student's paired t-test**							
	**ARTEMIS**	**Best competitor**	**US-align**	**RMalign**	**STAR3D**	**ARTS**	**Rclick**
**ARTEMIS**	*	0	0	0	0	0	0
**Best competitor**	0	*	0	0	0	0	0
**US-align**	0	0	*	0	0	0	0
**RMalign**	0	0	0	*	0	0	0
**STAR3D**	0	0	0	0	*	0	0
**ARTS**	0	0	0	0	0	*	5.69E-108
**Rclick**	0	0	0	0	0	5.69E-108	*
**Wilcoxon signed-rank test**							
	**ARTEMIS**	**Best competitor**	**US-align**	**RMalign**	**STAR3D**	**ARTS**	**Rclick**
**ARTEMIS**	*	0	0	0	0	0	0
**Best competitor**	0	*	0	0	0	0	0
**US-align**	0	0	*	0	0	0	0
**RMalign**	0	0	0	*	0	0	0
**STAR3D**	0	0	0	0	*	0	0
**ARTS**	0	0	0	0	0	*	2.39E-113
**Rclick**	0	0	0	0	0	2.39E-113	*

Supplementary Table S4. Student's paired t-test and Wilcoxon signed-rank test p-values calculated for each pair of the superposition tools based on the sets of TM-score$_{RNA}$ values, as measured on the dataset of 637 RNA chains in building topology-independent alignments. Zeros correspond to values < 1e-303.

**Student's paired t-test**				
	**ARTEMIS**	**Best competitor**	**US-align**	**Rclick**
**ARTEMIS**	*	0	0	0
**Best competitor**	0	*	0	0
**US-align**	0	0	*	1.10E-03
**Rclick**	0	0	1.10E-03	*
**Wilcoxon signed-rank test**				
	**ARTEMIS**	**Best competitor**	**US-align**	**Rclick**
**ARTEMIS**	*	0	0	0
**Best competitor**	0	*	0	0
**US-align**	0	0	*	0
**Rclick**	0	0	0	*

Supplementary Table S5. Absolute differences in TM-score$_{RNA}$ values between ARTEMIS and US-align and between ARTEMIS and the best competitor on the large benchmark dataset.

Alignment	sequentially-ordered		topology-independent		best of two	
Range	$TM_{ARTEMIS} - TM_{USalign}$	$TM_{ARTEMIS} - TM_{BestTool}$	$TM_{ARTEMIS} - TM_{USalign}$	$TM_{ARTEMIS} - TM_{BestTool}$	$TM_{ARTEMIS} - TM_{USalign}$	$TM_{ARTEMIS} - TM_{BestTool}$
X > 0.5	18	0	4	0	4	0
0.4 < X ≤ 0.5	17	1	11	0	11	0
0.3 < X ≤ 0.4	35	0	21	0	17	0
0.2 < X ≤ 0.3	109	8	394	36	337	25
0.1 < X ≤ 0.2	5109	1071	22500	3453	20714	2543
0.0 < X ≤ 0.1	247658	236138	286174	262636	240582	224222
X = 0.0	3501	4219	3258	5059	3067	4641
-0.1 ≤ X < 0.0	81334	96337	91173	132223	71965	105139
-0.2 ≤ X < -0.1	48	55	1580	1708	1135	1262
-0.3 ≤ X < -0.2	3	3	17	17	0	0
-0.4 ≤ X < -0.3	0	0	0	0	0	0
-0.5 ≤ X < -0.4	0	0	0	0	0	0
X < -0.5	0	0	0	0	0	0

Supplementary Table S6. The list of 99 backbone-permuted structural similarities. Structure identifiers are built by concatenating PDB entries and chain identifiers, separated with dots. Seven pairs featuring the helical packing motif are underscored.

6cu1.A 6t4q.6	5v93.a 5z3g.A	6cu1.A 2zue.B	6cu1.A 6ugg.A	6cu1.A 2nre.F
6cu1.A 6r87.B	5hd1.2a 3j7y.A	6cu1.A 3a2k.C	6cu1.A 4lck.B	6cu1.A 3akz.E
6cu1.A 5o2r.x	6cu1.A 6t4q.7	6cu1.A 6rfl.U	6cu1.A 5x6b.P	6cu1.A 4v8n.AW
6cu1.A 6t7t.6	6cu1.A 4v9i.AY	6cu1.A 3wc1.P	6cu1.A 3wqy.C	5tpy.A 5t5a.A
6cu1.A 2der.C	6cu1.A 4yye.C	5on2.B 6cu1.A	6cu1.A 3eph.E	6cu1.A 1u0b.A
6cu1.A 6r5q.2	6cu1.A 6ip5.zu	6cu1.A 2du3.D	6cu1.A 5el6.3K	3am1.B 6cu1.A
6cu1.A 4wj4.B	6cu1.A 4v7l.AY	6jxm.B 6cu1.A	1wz2.C 6cu1.A	6cu1.A 1g59.B
6cu1.A 6q9a.7	5u4j.a 2a64.A	6cu1.A 3j46.p	5ah5.D 6cu1.A	1s03.A 1i6u.C
6cu1.A 1qf6.B	6cu1.A 5wwr.C	6cu1.A 5e6m.C	6cu1.A 1h4s.T	1u9s.A 3ndb.M
6cu1.A 6tbv.PTR1	4v8d.AB 6cu1.A	6cu1.A 4rdx.C	6cu1.A 5lzs.ii	3w1k.F 6cu1.A
6cu1.A 6tb3.n	6gaw.BA 4v4a.AA	2zzm.B 6cu1.A	6cu1.A 2fk6.R	6dmc.A 3iwn.A
6c4h.A 6chr.A	3q1q.C 6cu1.A	6cu1.A 5b63.D	6cu1.A 4v5l.AY	3iwn.A 5t83.A
6cu1.A 6ufm.A	6cu1.A 6cae.1y	6cu1.A 6q97.7	6cu1.A 2d6f.F	6dvk.H 3iwn.A
6cu1.A 4v6u.A0	6cu1.A 1il2.C	4v8b.AB 6cu1.A	3dil.A 3pdr.A	3iwn.A 5u3g.B
6cu1.A 5j8b.x	6cu1.A 5vpp.QV	6cu1.A 4v6u.A1	6cu1.A 4v5g.AY	4jf2.A 2j28.8
6cu1.A 2dr2.B	6cu1.A 6v3a.v	6cu1.A 2dlc.Y	6cu1.A 5ud5.D	3j7y.A 5t7v.A
6cu1.A 3amt.B	6cu1.A 1gax.C	6cu1.A 1jgq.D	6cu1.A 2iy5.T	5mmm.a 3j7y.A
6cu1.A 6i0y.V	6cu1.A 6az1.2	6ah3.T 6cu1.A	6cu1.A 1ser.T	5xyi.2 3j7y.A
6cu1.A 3j7a.7	5g2x.A 6c4h.A	1h3e.B 6cu1.A	6cu1.A 3icq.D	6cu1.A 6qdw.v
6cu1.A 1j1u.B	6cu1.A 2csx.C	6cu1.A 6ek0.S6	5lzd.y 6cu1.A	

Supplementary Table S7. Performance of the topology-independent superposition tools on tRNA-like structures in RMSD and coverage values.

Tool				ARTEMIS		US-align		Rclick		RNAhugs GEOS		RNAhugs GENS	
**tRNA vs.**	**PDB id Chain**	**Length**	**Permutation**	**RMSD**	**tRNA Cov.**	**RMSD**	**tRNA Cov.**	**RMSD**	**tRNA Cov.**	**RMSD**	**tRNA Cov.**	**RMSD**	**tRNA Cov.**
	1ivsC	75											
tmRNA fragment	2czjB	62	no	3.650	0.573	2.820	0.520	2.630	0.507	3.030	0.560	2.720	0.507
tRNA-Sec	3addC	88	no	3.180	0.853	3.020	0.853	3.010	0.813	3.020	0.853	3.020	0.853
TYMV tRNA-like	4p5jA	83	non-circular	3.190	0.840	3.060	0.867	3.430	0.853	3.150	0.867	3.270	0.867
Y RNA	6cu1A	79	circular	2.690	0.907	2.400	0.907	2.600	0.907	2.460	0.907	2.450	0.907
BMV tRNA-like	7samA	169	non-circular	4.150	0.853	4.260	0.707	4.180	0.880	4.050	0.760	3.550	0.587
**Mean**				3.372	0.805	3.112	0.771	3.170	0.792	3.142	0.789	3.002	0.744

Supplementary Table S8. Comparison of different states of the tRNA-like structure from Brome Mosaic Virus using ARTEMIS in -*superonly* mode.

**TM-score$_{RNA}$**			
	7SAM_A	7SC6_C	7SCQ_C
Isolated State, PDB entry 7SAM, chain A	-	0.687	0.630
Bound State 1, PDB entry 7SC6, chain C	0.687	-	0.781
Bound State 2, PDB entry 7SCQ, chain C	0.630	0.781	-
**RMSD (Å)**			
	7SAM_A	7SC6_C	7SCQ_C
Isolated State, PDB entry 7SAM, chain A	-	14.24	14.07
Bound State 1, PDB entry 7SC6, chain C	14.24	-	3.26
Bound State 2, PDB entry 7SCQ, chain C	14.07	3.26	-

Supplementary Table S9. Student's paired t-test and Wilcoxon signed-rank test p-values calculated for each pair of the superposition tools based on the sets of TM-score$_{RNA}$ values, as measured on the dataset of 16 RNA chains in building topology-independent alignments.

**Student's paired t-test**						
	**ARTEMIS**	**Best competitor**	**US-align**	**Rclick**	**RNAhugs GEOS**	**RNAhugs GENS**
**ARTEMIS**	*	5.46E-05	3.56E-32	1.21E-27	6.57E-29	1.53E-85
**Best competitor**	5.46E-05	*	2.58E-30	1.38E-26	6.26E-22	3.88E-81
**US-align**	3.56E-32	2.58E-30	*	1.12E-11	5.34E-08	7.5E-60
**Rclick**	1.21E-27	1.38E-26	1.12E-11	*	1.42E-01	2.75E-73
**RNAhugs GEOS**	6.57E-29	6.26E-22	5.34E-08	1.42E-01	*	2.85E-75
**RNAhugs GENS**	1.53E-85	3.88E-81	7.5E-60	2.75E-73	2.85E-75	*
**Wilcoxon signed-rank test**						
	**ARTEMIS**	**Best competitor**	**US-align**	**Rclick**	**RNAhugs GEOS**	**RNAhugs GENS**
**ARTEMIS**	*	1.36E-04	5.2E-29	6.46E-27	2.11E-29	4.02E-41
**Best competitor**	1.36E-04	*	3.53E-28	1.07E-25	3.82E-28	1.81E-40
**US-align**	5.2E-29	3.53E-28	*	3.72E-09	4.15E-07	1.13E-39
**Rclick**	6.46E-27	1.07E-25	3.72E-09	*	3.46E-01	2.42E-40
**RNAhugs GEOS**	2.11E-29	3.82E-28	4.15E-07	3.46E-01	*	6.38E-41
**RNAhugs GENS**	4.02E-41	1.81E-40	1.13E-39	2.42E-40	6.38E-41	*
